# Distinct motifs in the E protein are required for SARS-CoV-2 virus particle formation and lysosomal deacidification in host cells

**DOI:** 10.1128/jvi.00426-23

**Published:** 2023-10-13

**Authors:** Koya Miura, Youichi Suzuki, Kotaro Ishida, Masashi Arakawa, Hong Wu, Yoshihiko Fujioka, Akino Emi, Koki Maeda, Ryusei Hamajima, Takashi Nakano, Takeshi Tenno, Hidekazu Hiroaki, Eiji Morita

**Affiliations:** 1 Department of Biochemistry and Molecular Biology, Faculty of Agriculture and Life Science, Hirosaki University, Aomori, Japan; 2 Department of Microbiology and Infection Control, Faculty of Medicine, Osaka Medical and Pharmaceutical University, Osaka, Japan; 3 Laboratory of Structural and Molecular Pharmacology, Graduate School of Pharmaceutical Sciences, Nagoya University, Aichi, Japan; 4 BeCellBar LLC, Nagoya, Aichi, Japan; St. Jude Children's Research Hospital, Memphis, Tennessee, USA

**Keywords:** severe acute respiratory syndrome coronavirus 2 (SARS-CoV-2), virus-like particle (VLP), envelope (E) protein, pH indicator, lysosomal pH, virion secretion, HiBiT tag, PDZ domain protein, ESCRT pathway, PDZ-binding motif

## Abstract

Severe acute respiratory syndrome-coronavirus-2 (SARS-CoV-2) is a major public health concern, but the mechanisms underlying its viral particle formation are not well understood. In this study, we established a system for producing virus-like particles (VLPs) by expressing four structural proteins that make up SARS-CoV-2 virus particles in cells and used a spike (S) protein fused with the HiBiT peptide as a marker for evaluating VLP production. Using this system, we confirmed that the E protein plays an important role in VLP release. Both the co-expression of VPS4A K173Q and ORF3A and treatment with bafilomycin A1 enhanced VLP release. These results suggest that VLPs are released in an endosomal sorting complex required for transport-independent manner and that lysosomal dysfunction is required for the efficient release of VLPs. Screening various E protein mutants revealed that the F56/Y57/Y59 amyloidization motif and the D72/L73/L74/V75 PDZ-binding motif (PBM) are critical for E protein function in VLP release. We also found that E protein expression led to an increase in the pH of lysosomes and that the N15 residue required for viroporin activity, the C40/C43 consensus sequence, or the K63 dibasic motif are required for its function. However, amyloidization or PBM mutations did not affect lysosomal deacidification, suggesting that the mechanisms of E protein activity during VLP formation and lysosomal deacidification are distinct. Overall, this study highlights the importance of the E protein in SARS-CoV-2 viral particle formation, and the results may be useful in the development of drugs that inhibit this process.

**IMPORTANCE:**

Severe acute respiratory syndrome-coronavirus-2 (SARS-CoV-2), the virus responsible for coronavirus disease 2019 (COVID-19), has caused a global public health crisis. The E protein, a structural protein found in this virus particle, is also known to be a viroporin. As such, it forms oligomeric ion channels or pores in the host cell membrane. However, the relationship between these two functions is poorly understood. In this study, we showed that the roles of E protein in virus particle and viroporin formation are distinct. This study contributes to the development of drugs that inhibit SARS-CoV-2 virus particle formation. Additionally, we designed a highly sensitive and high-throughput virus-like particle detection system using the HiBiT tag, which is a useful tool for studying the release of SARS-CoV-2.

## INTRODUCTION

The coronavirus disease 2019 (COVID-19) pandemic, caused by the severe acute respiratory syndrome coronavirus 2 (SARS-CoV-2), has had a global impact since its emergence in Wuhan, China, in December 2019. According to the World Health Organization, as of October 2022, the virus has caused 600 million confirmed cases and six million deaths worldwide. Coronaviruses, including SARS-CoV-1 and MERS-CoV, are known to cause severe and often fatal diseases (1). Currently, drugs targeting the RNA-dependent polymerase or the protease enzymes of SARS-CoV-2 are effective treatments for SARS-CoV-2 infections (2). However, it is necessary to develop drugs with alternative modes of action that can effectively treat the virus and have minimal side effects.

SARS-CoV-2 is a member of the β-coronavirus (β-CoV) family and has a single-stranded (+) RNA genome of approximately 30 kb (3). It has 79.6% sequence identity with SARS-CoV-1, and both viruses enter cells using the angiotensin-converting enzyme 2 (ACE2) receptor, which is present on the target cell surface (4, 5). Upon entry of SARS-CoV-2 into the host cells, RNA from the viral genome is synthesized in the cytoplasm (6, 7). Following the synthesis of the viral RNA genome and protein, four structural proteins, the spike (S), nucleocapsid (N), membrane (M), and envelope (E) proteins, assemble on the membrane with the genomic RNA to form a new virion (8 – 10). The S protein, which binds to the ACE2 receptor, is important for viral entry into the host cells (11). The N protein of SARS-CoV-2 binds to and protects the genomic RNA and promotes virion assembly and maturation through its interaction with the C-terminus of the M protein (12, 13). The M protein is involved in the assembly of structural proteins and the formation of viral particles in the endoplasmic reticulum (ER)-Golgi intermediate compartment, and it is the most abundant protein in viral particles (12, 14). The function of the E protein in the assembly and budding of SARS-CoV-2 remain unclear, but research on SARS-CoV-1 and SARS-CoV-2 has demonstrated that it has a crucial role in virion formation, as the titers of viruses lacking the E protein are greatly reduced compared to those of wild-type viruses (15 – 17). Studies of SARS-CoV-2 virus-like particles (VLPs) have also shown that the E protein plays a role in modulating the secretory pathway of the S protein (18). The interactions among these four structural proteins are crucial for the proper assembly, genome packaging, and budding of progeny coronavirus particles (12). After virion assembly, β-CoVs, including SARS-CoV-2, utilize lysosomal trafficking to release their progeny particles rather than the biosynthetic secretory pathway commonly used by other enveloped viruses (19). This process can lead to lysosomal deacidification, the inactivation of lysosomal enzymes, and disruption of antigen presentation pathways (19).

The SARS-CoV-2 E protein is a single transmembrane protein composed of 75 amino acids, and its sequence is similar to that of SARS-CoV-1 (20). The E protein is a structural coronavirus protein that is incorporated into virions in small amounts (12). The E protein can promote virus particle assembly by cooperating with the M protein (18, 21 – 26). Additionally, the E protein has been reported to function as a viroporin, with monovalent cation and Ca^2+^-selective ion channel activity, through self-assembly into a pentameric structure (6, 16, 27 – 32). Studies of SARS-CoV-1 have shown that its activity is not essential for viral production, but it can enhance viral pathogenesis by modulating cytokine production (16). The C-terminus of coronaviral E proteins contains a PDZ-binding motif (PBM) sequence that interacts with host PDZ domain proteins, such as PALS1 and syntenin1 (33 – 39). The PDZ domain is a protein-protein interaction module found in a variety of species that is involved in various biological processes, including protein transport, cell adhesion, ion channel formation, and signal transduction (40). Proteome analysis has been used to identify several other PDZ domain proteins, including ZO-1, PALS2, and PTPN13, that may interact with the E protein (41), suggesting that multiple PDZ domain proteins are involved in E protein function. Although the infectivity of recombinant SARS-CoV-1 with a mutation in the PBM of the E protein does not significantly impact viral propagation, studies have shown that deletion of the entire PBM region in the SARS-CoV-1 E protein or mutations in the E proteins of SARS-CoV-2 or MERS-CoV affects viral propagation (15, 42). These findings strongly suggest that the interaction between the host PDZ domain proteins and E proteins plays a role in viral propagation. However, the specific roles of these interactions in the viral life cycle remain unclear.

Previous studies have shown that VLPs can be efficiently produced when all four structural proteins (S, M, N, and E) are expressed. In this study, we fused the HiBiT tag, a highly sensitive detection tag (43), to the C-terminus of the S protein and used HiBiT activity measurements as an extremely sensitive method for quantifying VLPs. Furthermore, we monitored changes in lysosomal acidification caused by the expression of the E and ORF3A proteins and evaluated how these proteins affected viroporin function. Our study revealed a relationship between viral particle formation and viroporin function in SARS-CoV-2 VLP-producing cells.

## RESULTS

### Establishment of a highly sensitive SARS-CoV-2 virus-like particle detection system

**Fig 1:**
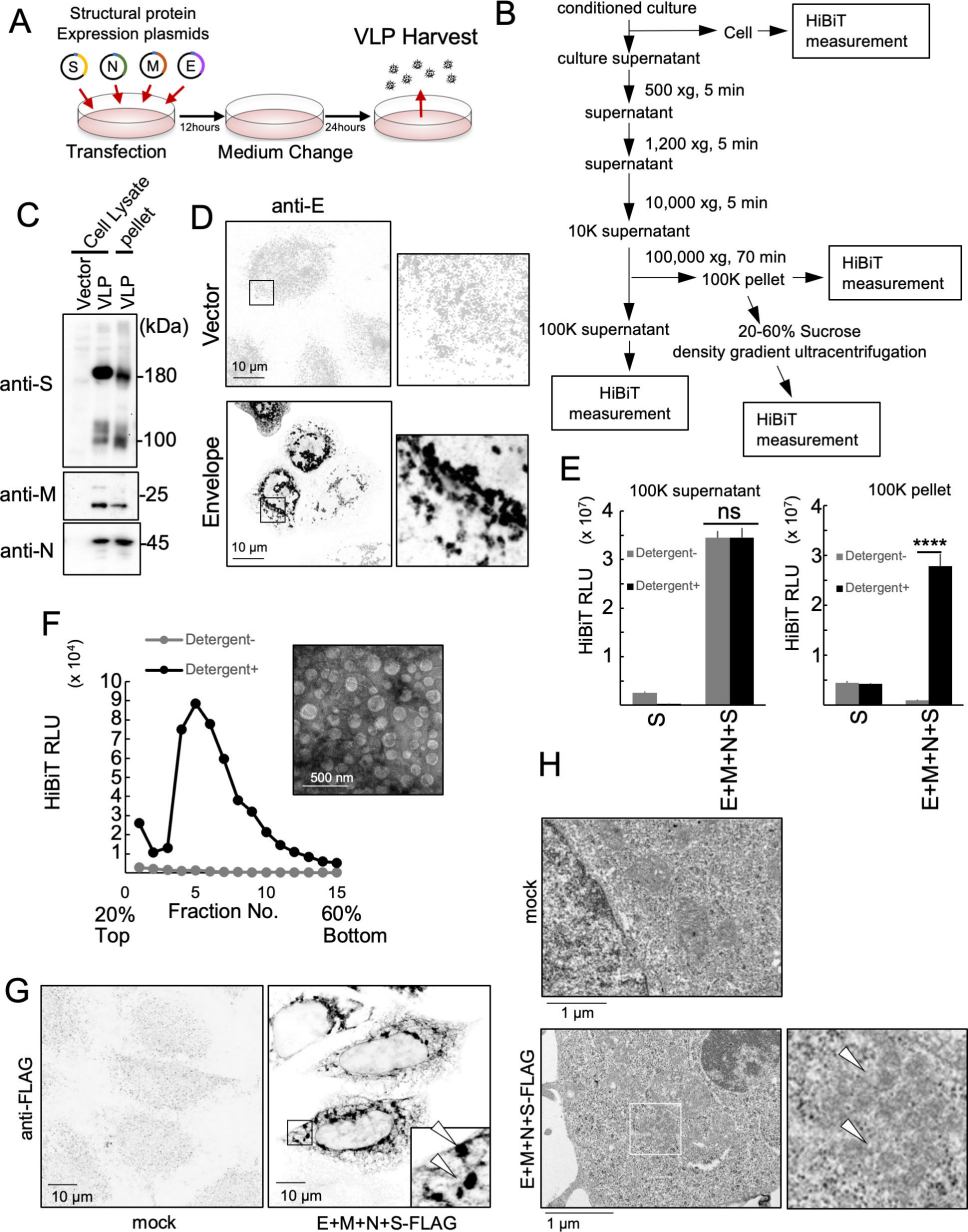
Highly sensitive SARS-CoV-2 virus-like particle detection system. (**A**) Schematic illustration of the SARS-CoV-2 VLP-producing system. (**B**) Flowchart of VLP purification. (**C**) Detection of S, M, and N proteins from the cells producing VLPs and the purified VLP fraction. Thirty-six hours after transfection, whole-cell lysate (WCL) was prepared from the cells transfected with an empty vector (lane 1) or the S-FLAG-HiBiT, M, N, and E protein-expressing vector (lane 2). The WCL and VLP fraction (100,000 × *g* pellet fraction, lane 3) was separated using sodium dodecyl sulfate polyacrylamide electrophoresis (SDS-PAGE) and detected using the S-FLAG-HiBiT (top panel), M protein (middle panel), N protein (bottom panel), and their specific antibodies. (**D**) Detection of E protein from a VLP-expressing cell. Twenty-four hours after transfection, an empty vector (upper) or E protein-expressing vector (lower) transfected HeLa cells were fixed and stained with anti-E antibodies. Magnified images are shown in the right panels. Bar = 10 µm. (**E**) HiBiT activities in the 100,000 × *g* supernatant fraction (left panel) and 100,000 × *g* pellet fraction (right panel). Thirty-six hours after transfection, culture supernatants from the HEK293T cells expressing S-FLAG-HiBIT protein only or the S-FLAG-HiBiT, M, N, and E proteins were fractionated using serial centrifugation. HiBiT activities in the indicated fractions were measured with (black bars) or without (gray bars) 0.1% (vol/vol) TritonX-100. ****P < 0.0001; ns, not significant (Tukey’s *t-*test). (**F**) Sucrose density gradient (20%–60%) ultracentrifugation assay of the 100,000 × *g* pellet fraction from culture supernatants of 293T cells expressing the S-FLAG-HiBiT, M, N, and E proteins. HiBiT activities in each fraction were measured with (black line) or without (gray line) 0.1% (vol/vol) TritonX-100. Negatively stained transmission electron microscope images of fractions no. 4–6 are shown in the right upper panel. Bar = 500 nm. (**G**) Subcellular localization of the S-FLAG-HiBiT protein and a TEM image of VLP-expressing cells. Twenty-four hours after transfection, HeLa cells expressing the S-FLAG-HiBiT, M, N, and E proteins (left bottom panel) or the mock control (left upper panel) were stained using anti-FLAG antibodies. The arrowhead indicates a VLP-specific structure. Bar = 10 µm. (**H**) Ninety-six hours after transfection, HEK293T cells expressing the S-FLAG-HiBiT, M, N, and E proteins (bottom) or mock-transfected negative control cells (top) were fixed, ultrathin sectioned, stained, and observed by TEM. Magnified images are shown in the bottom right panel. The arrowhead indicates a VLP-like structure. Bar = 1 µm.

To learn more about the mechanism of SARS-CoV-2 particle formation, we focused on studying the VLPs that can be produced by inducing the expression of viral structural proteins in cells. In our study, we used HEK293T cells transfected with the four SARS-CoV-2 structural proteins. Thirty-six hours after transfection, we collected the VLPs from the culture supernatants (Fig. 1A). The collected medium was then centrifuged at three different speeds (500 × *g;* 1,200 × *g*; and 10,000 × *g*) for 5 min each to perform crude purification and remove cell debris. Further purification of VLPs was performed using ultracentrifugation at 100,000 × *g*, and the pellet fraction contained the VLPs (Fig. 1B). Western blotting was used to confirm that the purified VLPs contained the structural proteins S, M, and N (Fig. 1C). E protein expression was confirmed using fluorescent immunostaining, as western blotting was unsuccessful (Fig. 1D). To quantify the VLPs, we used a HiBiT tag (a Split NanoLuc fused to the C-terminus of the spike protein), which enabled extremely sensitive measurements. The C-terminus of the spike protein is located within the VLP, which allowed us to evaluate its status in the culture supernatant by comparing the HiBiT activity in the presence or absence of detergent. We observed a significant detergent-dependent increase in HiBiT activity in the 100,000 × *g* pellet when all structural proteins were present (Fig. 1E, right). However, we did not observe a detergent-dependent increase in HiBiT activity in the 100,000 × *g* supernatant despite the presence of all four structural proteins (Fig. 1E, left). These results suggested that all S proteins in the 100,000 × *g* pellet fraction in culture supernatant are released as membrane-enveloped VLPs. We named this detection system the Detergent Assay and used it in subsequent experiments. Because the VLPs released into extracellular environment appeared to be enveloped by membranes, the status of the VLPs in the 100,000 × *g* precipitate was investigated using 20%–60% sucrose density gradient ultracentrifugation. The HiBiT activity of each fraction was measured in the presence or absence of detergents. After the fractions were treated with detergent, we detected a peak in HiBiT activity, especially in the heavy fraction (Fig. 1F). The presence of 100 nm vesicles in these fractions supports the conclusion that they represent VLP-positive fractions. These results suggest that VLPs are released into the extracellular environment as enveloped VLPs. Immunofluorescence imaging was also performed to evaluate the intracellular status of VLPs when all four structural proteins are expressed (Fig. 1G, right). These analyses revealed the assembly of S-protein-positive structures in the cytoplasm. TEM of ultra-thin sections of cells expressing VLPs revealed the formation of approximately 100 nm particles in the cytoplasm (Fig. 1H, right, arrows), which were not observed in mock-transfected HEK293T cells. These results suggest that the formation of VLPs in these cells was similar to that observed during SARS-CoV-2 infection.

### Requirement of SARS-CoV-2 structural proteins for VLP formation

**Fig 2:**
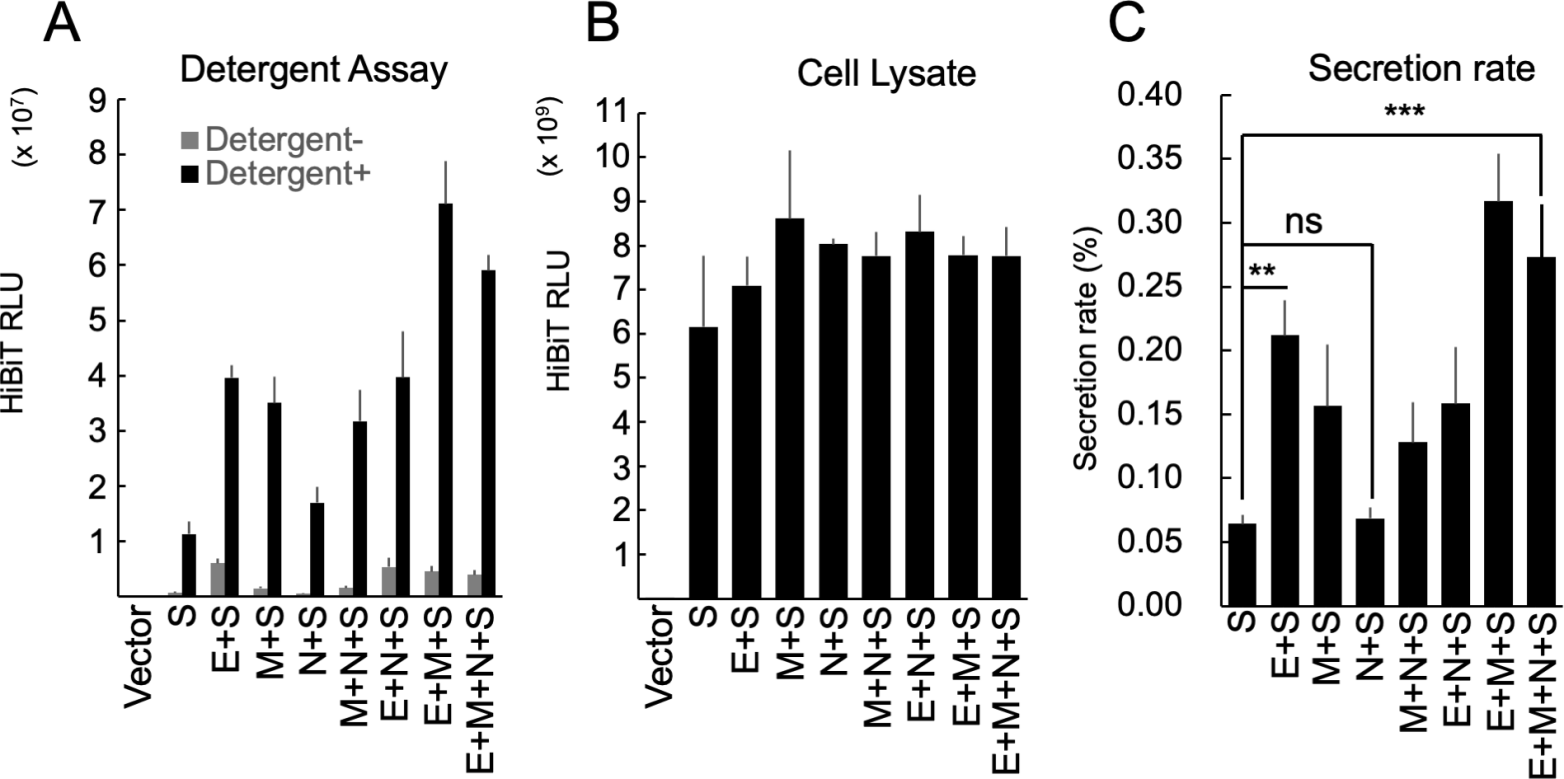
Requirement of SARS-CoV-2 structural proteins for VLP release. (**A**) HiBiT activities in the 100,000 × *g* pellet fraction of culture supernatants derived from HEK293T cells expressing various combinations of structural proteins. Thirty-six hours after transfection, the VLPs in culture supernatants from the HEK293T cells expressing the indicated combination of structural proteins were purified using serial centrifugation. HiBiT activities in the 100,000 × *g* pellet fractions were measured with (black bars) or without (gray bars) 0.1% (vol/vol) TritonX-100. (**B**) HiBiT activities in the WCL fraction from the experiment (**A**). (**C**) Secretion rate of S-FLAG-HiBiT. The secretion rate was calculated by dividing the culture supernatant HiBiT value by the total HiBiT value of the cell culture. **P < 0.01; ***P < 0.001; ns not significant (Tukey’s *t-*test).

Various structural protein combinations were expressed in the experimental cells to determine the minimum structural protein requirements for VLP production. The HiBiT activity in culture supernatants and cell lysates was measured, and the secretion rate was calculated by dividing the supernatant HiBiT activity by the total HiBiT activity (Fig. 2A through C). The results showed that the presence of the E protein significantly increased secretion compared to that of the S protein alone. The presence of the M protein also increased secretion, but the presence of the N protein had no effect on secretion. The co-expression of E + N or M + N did not change secretion compared to that of E or M alone, but the co-expression of E + M led to higher secretion. This suggests that the E and M proteins are crucial for VLP production. The E protein helps form virions in coronaviruses (44), and previous reports have indicated that only a small amount is incorporated into virus particles (45). Therefore, we conducted further experiments to explore the role of the E protein in virus particle formation.

### The endosomal sorting complexes required for transport pathway is not involved in SARS-CoV-2 VLP release

**Fig 3:**
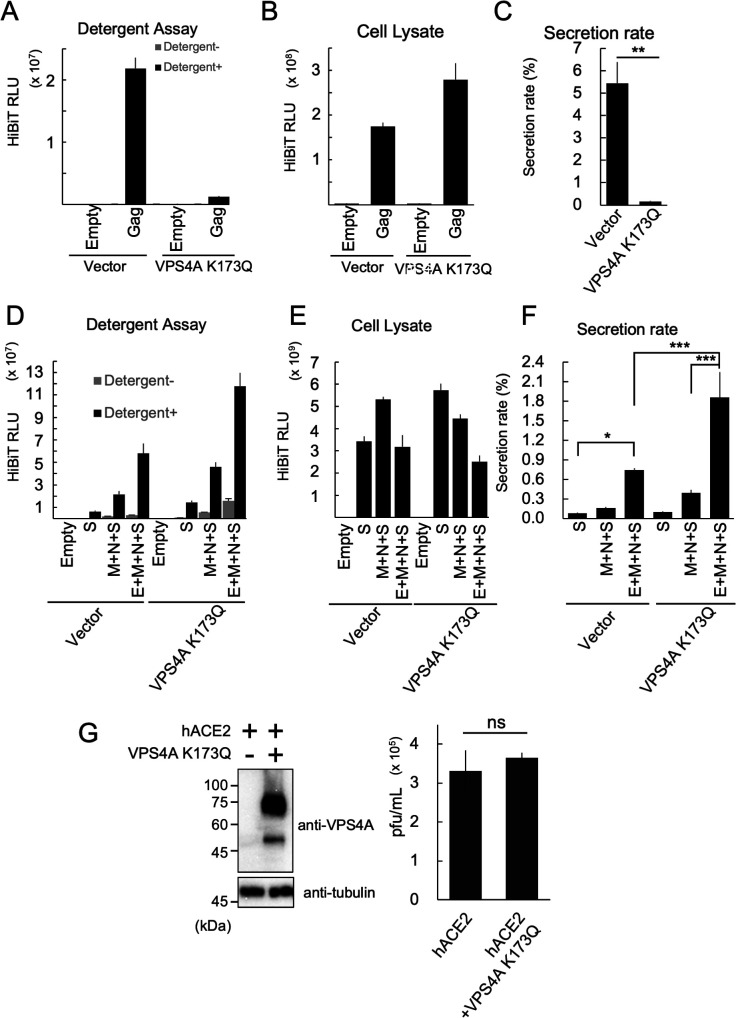
ESCRT pathway is not involved in SARS-CoV-2 VLP release. (**A–C**) Effects of the ESCRT pathway on Gag protein secretion. (**A**) HiBiT activities in the 20,000 × *g* pellet fraction of the culture supernatants derived from HEK293T cells expressing HIV-Gag-HiBiT. Thirty-six hours after transfection, the VLPs in the culture supernatants from the HEK293T cells expressing HIV-Gag-HiBiT with or without VPS4A K173Q were purified using serial centrifugation. HiBiT activities in the 20,000 × *g* pellet fraction were measured with (black bars) or without (gray bars) 0.1% (vol/vol) TritonX-100. (**B**) HiBiT activities in the WCL fraction of experiment (**A**). (**C**) Secretion rate of HIV-Gag-HiBiT. The secretion rate was calculated by dividing the culture supernatant HiBiT value by the total HiBiT value for the cell culture. *P < 0.05, ***P < 0.001 (Tukey’s *t-*test). (**D–F**) Effects of the ESCRT pathway on SARS-CoV-2 VLP release. (**D**) HiBiT activities in the 100,000 × *g* pellet fraction of the culture supernatants derived from HEK293T cells expressing various combinations of SARS-CoV-2 structural proteins. Thirty-six hours after transfection, the VLPs in the culture supernatants from the HEK293T cells expressing the indicated SARS-CoV-2 structural proteins expressed with or without VPS4A K173Q were purified using serial centrifugation. HiBiT activities in the 100,000 × *g* pellet fraction were measured with (black bars) or without (gray bars) 0.1% (vol/vol) TritonX-100. (**E**) HiBiT activities in the WCL fraction from experiment (**D**). (**F**) Secretion rate of SARS-CoV-2 S-FLAG-HiBiT. The secretion rate was calculated by dividing the culture supernatant HiBiT value by the total HiBiT value in cell culture. *P < 0.05, ***P < 0.001, (Tukey’s *t-*test). (**G**) Effects of the ESCRT pathway on SARS-CoV-2 propagation. HEK293T cells were co-transfected with a vector expressing human ACE2 (hACE2) and either a VPS4A K173Q expressing vector or an empty vector. After 24 h of culture, the cells were infected with SARS-CoV-2 at an MOI (multiplicity of infection) of 0.1. The expression levels of VPS4A K173Q (top left) and alpha-tubulin (bottom left), as well as the SARS-CoV-2 infectious titer (right graph), were measured at 48 h post infection (hpi).

The endosomal sorting complex required for transport (ESCRT) pathway is known to be involved in the budding of some enveloped viruses, including retroviruses (46, 47). To determine whether SARS-CoV-2 virus particle budding depends on this pathway, we conducted experiments using VLPs. VPS4A is a member of the AAA-ATPase family of proteins that forms a hexameric complex. This protein complex plays a crucial role in the disassembly of ESC-RT factors. Overexpression of ATP hydrolysis-deficient mutations in VPS4A effectively inhibits the ESCRT pathway (48). We performed an ESCRT pathway inhibition experiment using the VPS4A K173Q dominant-negative mutant to block the release of the human immunodeficiency type I (HIV-I) Gag protein, which is known to bud through the ESCRT pathway (48). The Detergent Assay showed that the released Gag protein was enveloped by the membrane (Fig. 3A). The HiBiT activity of the cell lysates was also measured (Fig. 3B), and the secretion rate was calculated, revealing a dramatic decrease in Gag secretion due to VPS4A K173Q expression (Fig. 3C). A similar experiment was conducted using SARS-CoV-2 VLPs to examine the effect of the E protein on the ESCRT pathway. The secretion of the S protein alone (S), without the E protein (M + N + S), and with all structural proteins (E + M + *N* + S) was measured. As expected, the secretion of E + M + *N* + S was significantly higher than that of M + N + S when the ESCRT pathway was not inhibited (Fig. 3D through F). However, when the ESCRT pathway was inhibited, we did not observe a decrease in the secretion of the S protein alone, M + N + S, or E + M + N + S. Instead, inhibiting the ESCRT pathway led to a significant increase in VLP secretion (Fig. 3D through F), indicating that SARS-CoV-2 VLPs undergo ESCRT-independent budding. To assess the effects of VPS4A K173Q expression on the SARS-CoV-2 viral life cycle, we conducted SARS-CoV-2 infection studies. A previous study has shown that the exogenous overexpression of hACE2 significantly increased the susceptibility of HEK293T cells to SARS-CoV-2 infection (49). As shown in Fig. 3G, VPS4A K173Q expression in these cells did not inhibit SARS-CoV-2 propagation; rather, it led to a slight increase. This observation also suggests that the ESCRT pathway does not play a significant role in the SARS-CoV-2 life cycle.

### Enhancement of VLP release by the inhibition of lysosomal acidification

**Fig 4:**
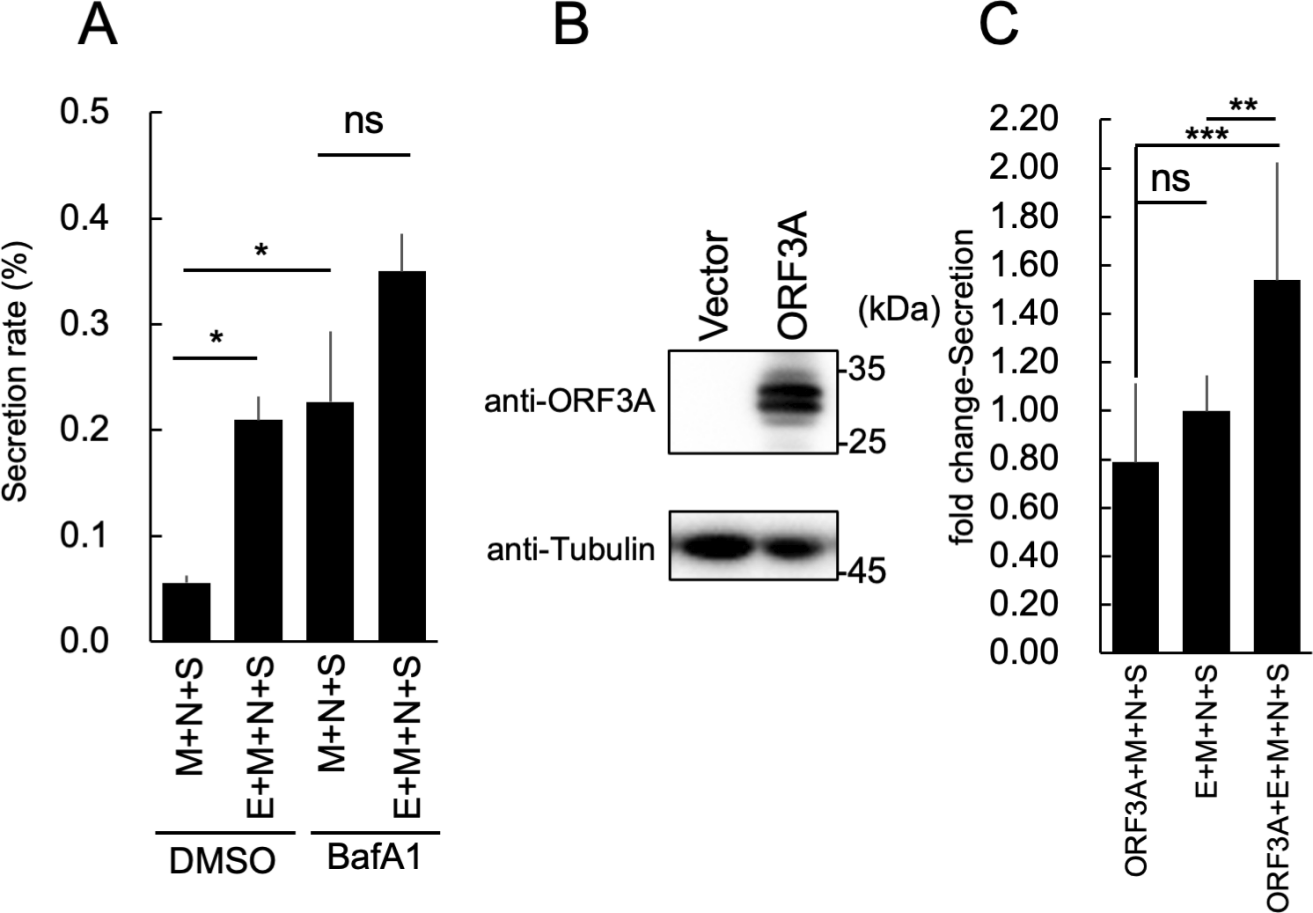
Enhancement of VLP release by the inhibition of lysosomal acidification. (**A**) Effect of bafilomycin A1 (BafA1) on VLP release. Thirty-six hours after transfection, the VLPs in culture supernatants from the HEK293T cells expressing the indicated SARS-CoV-2 structural proteins and treated with or without 50 nM of BafA1 were purified using serial centrifugation. HiBiT activities in the cells and supernatant fractions were measured. The secretion rate was calculated by dividing the culture supernatant HiBiT value by the total HiBiT value of the cell culture. After calculating the secretion rate, the fold-change secretion was determined based on that of cells expressing E + M + N + S. *P < 0.05; ns, not significant (Tukey’s *t-*test). (**B**) Expression of the SARS-CoV-2 ORF3A protein. SARS-CoV-2 ORF3A-expressing vectors were transfected into HEK293T cells. Thirty-six hours after transfection, the expression of ORF3A protein (upper panel) or alpha-tubulin (lower panel) was confirmed using western blotting and specific antibodies. (**C**) Effect of ORF3A protein expression on VLP release. Thirty-six hours after transfection, the VLPs in the culture supernatants from the HEK293T cells that expressed the indicated SARS-CoV-2 structural proteins with or without ORF3A protein were purified using serial centrifugation. HiBiT activities in the cells and supernatant fractions were measured. The secretion rate was calculated by dividing the culture supernatant HiBiT value by the total HiBiT value of the cell culture. *P < 0.05; ns, not significant (Tukey’s *t-*test). After calculating the secretion rate, the fold-change secretion was determined based on that of cells expressing E + M + *N* + S. **P < 0.01; ***P < 0.001; ns, not significant (Tukey’s *t-*test).

The inhibition of the ESCRT pathway impairs the delivery of lysosomal hydrolases (50), resulting in lysosomal dysfunction. We hypothesized that this dysfunction contributes to the increased release of SARS-CoV-2 VLPs. Furthermore, previous reports have shown that SARS-CoV-2 infection increases lysosomal pH (19). Therefore, we investigated the effect of reduced lysosomal pH on VLP production using bafilomycin A1 (BafA1), a V-ATPase inhibitor that inhibits lysosomal acidification (51). We found that E-dependent VLP release occurred after DMSO treatment, but that no E-dependent increase in VLP secretion occurred after BafA1 treatment (Fig. 4A). To further explore the role of lysosomal acidification in VLP production, we investigated the effects of ORF3A expression on VLP secretion. The ORF3A protein, a reported viroporin, has been shown to inhibit lysosome acidification (19, 52). The expression of ORF3A was confirmed using western blotting (Fig. 4B). The presence of both the E and ORF3A proteins resulted in the release of the highest amount of VLPs, which was approximately 1.5 times that of E + M + N + S alone (Fig. 4C). In contrast, there was no difference in the amount of VLPs released when E + M + N + S and ORF3A + M + N + S were compared (Fig. 4C). These results suggest that the E and ORF3A proteins exert a synergistic effect and support the hypothesis that both proteins aid VLP secretion by inhibiting lysosomal acidification.

### The functional domain of the E protein in VLP production

**Fig 5:**
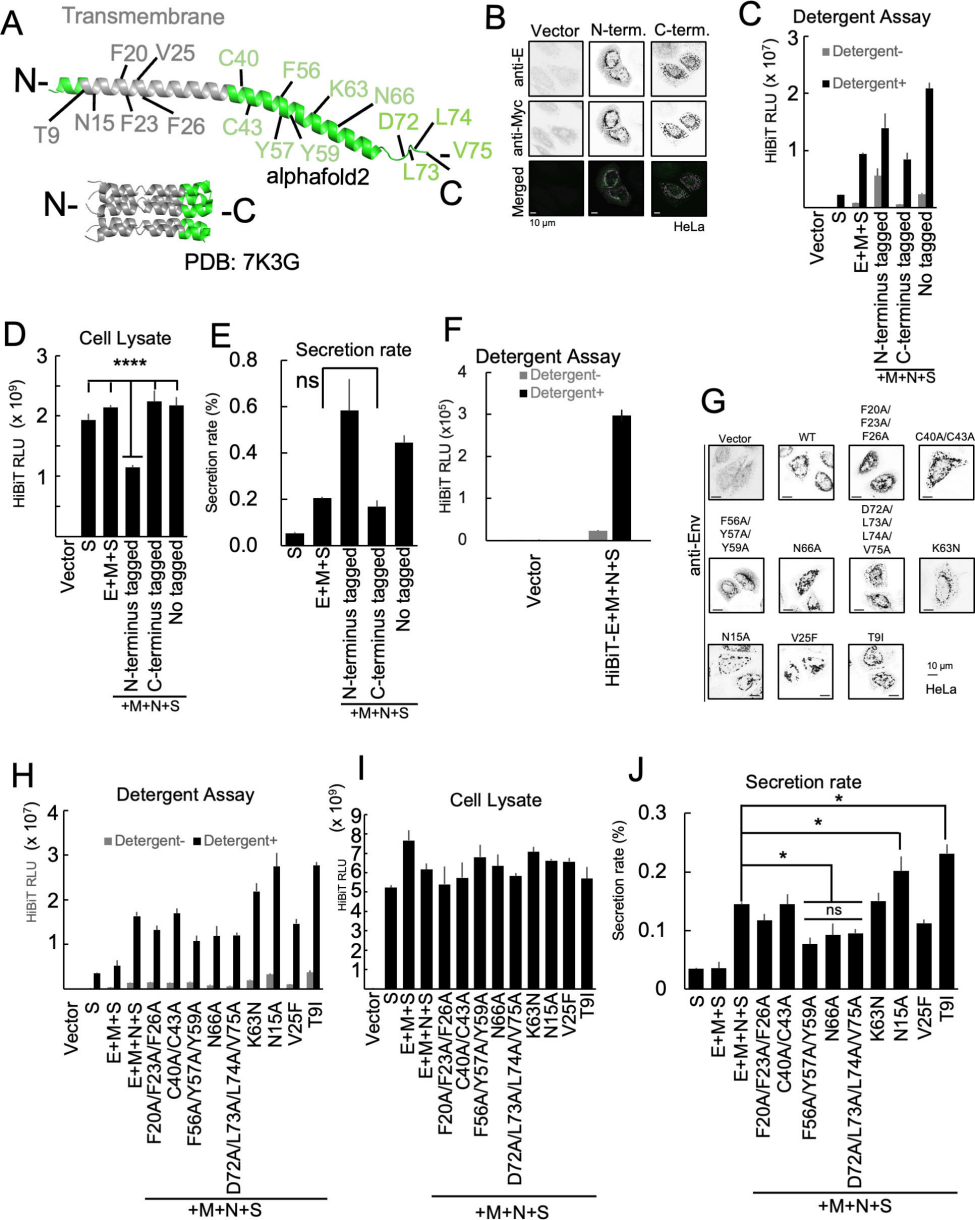
Functional domain of E protein required for VLP release. (**A**) The structure of the E protein. The model structure of E protein by alphafold2 (top) and the NMR structure of pentameric E protein (PDB:7K3G) (bottom). The amino acids residues this study focused on are indicated. The transmembrane region is shown in gray. Other regions of interest are shown in green. (**B**) Expression of Myc-tagged E proteins. HeLa cells were transfected with an empty vector (left panels), an expression vector for E protein fused with a Myc tag at the N-terminus (middle panels), or C-terminus (right panels). Twenty-four hours after transfection, the cells were stained with anti-E (green, upper panels) and anti-Myc (magenta, middle panels) antibodies. Merged images are shown in the bottom panels. Bar = 10 µm. (C, D, and E) Effect of Myc-tag fusion on VLP release. (**C**) HiBiT activities in the 100,000 × *g* pellet fractions of cell culture supernatants derived from HEK293T cells expressing various combinations of structural proteins. Thirty-six hours after transfection, the VLPs in the culture supernatants from the HEK293T cells that expressed the indicated combination of structural proteins were purified using serial centrifugation. HiBiT activities in the 100,000 × *g* pellet fractions were measured with (black bars) or without (gray bars) 0.1% (vol/vol) TritonX-100. (**D**) HiBiT activities in the WCL fraction of experiment (**C**). (**E**) Secretion rate of S-FLAG-HiBiT. The secretion rate was calculated by dividing the culture supernatant HiBiT value by the total HiBiT value in the cell culture. ****P < 0.0001; ns, not significant (Tukey’s *t-*test). (**F**) HiBiT activities in the 100,000 × *g* pellet fraction of culture supernatant derived from cells expressing HiBiT-E and other structural proteins. Thirty-six hours after transfection, culture supernatants from the HEK293T cells expressing an empty vector or the HiBiT-E, S, M, and N proteins were fractionated using serial centrifugation. HiBiT activities in the 100,000 × *g* pellet fractions were measured with (black bars) or without (gray bars) 0.1% (vol/vol) TritonX-100. (**G**) Expression of E mutant proteins. HeLa cells were transfected with an empty vector (upper left panel) or an expression vector for the mutant E protein. Twenty-four hours after transfection, the cells were stained with anti-E antibodies. Bar = 10 µm. (H, I, and J) Effect of E protein mutations on VLP release. (**H**) HiBiT activities in the 100,000 × *g* pellet fractions of culture supernatants derived from HEK293T cells expressing VLPs and different mutant E proteins. Thirty-six hours after transfection, the VLPs in the culture supernatants from the HEK293T cells that expressed the indicated combination of structural proteins were purified using serial centrifugation. HiBiT activities in the 100,000 × *g* pellet fractions were measured with (black bars) or without (gray bars) 0.1% (vol/vol) TritonX-100. (**I**) HiBiT activities in the WCL fraction of experiment (**H**). (**J**) Secretion rate of S-FLAG-HiBiT. The secretion rate was calculated by dividing the culture supernatant HiBiT value by the total HiBiT value for the cell culture. *P < 0.05; ns, not significant (Tukey’s *t-*test).

Next, we evaluated the effect of mutations in the E protein on VLP secretion and analyzed the relationship between E protein structure and VLP production. The E protein is a single transmembrane protein consisting of 75 amino acids and has 94.7% sequence homology with the E protein of SARS-CoV-1 (53). In both SARS-CoV-1 and SARS-CoV-2, viruses lacking the E protein have been shown to have significantly reduced titers, suggesting that the E protein is required for virus particle formation (15, 16). Therefore, we created mutants with amino acid substitutions based on the previously reported functions of the E protein and sequences that are conserved among coronaviruses (Fig. 5A) (15, 16, 20, 21, 42, 44, 54 – 61). In addition, to investigate the effect of fusing tags to the C-terminus or N-terminus of the E protein, we constructed a Myc-tagged mutant and confirmed its expression using immuno-staining (Fig. 5B). An examination of VLPs secreted from cells expressing the E proteins with either N-terminus or C-terminus tags showed that there was a decrease in the HiBiT value in the cell lysate upon expression of Myc-E (fused to the N-terminus) (Fig. 5C and D). The secretion rate of VLPs decreased significantly when the Myc tag was fused to the C-terminus of the E protein (E-Myc). The secretion rate was similar to that which occurred when the E protein was not expressed (Fig. 5E). These results indicated that fusing tags to the C-terminus of E protein affect VLP formation. To investigate the presence of E protein in VLPs, we co-expressed a HiBiT-fused E protein at the N-terminus with M, N, and S proteins. Notably, the HiBiT tag was not fused with the S protein in this case. As shown in Fig. 5F, HiBiT-E was detected in the culture supernatant, similar to S-HiBiT-, M-, N-, and E-expressing cells. Importantly, this HiBiT activity was only detected in the presence of detergent, suggesting that a portion of the E protein was, indeed, incorporated into these VLPs. Next, we observed the effects of various E-protein mutations on VLP production and release. T9I is a mutation in the E gene of the SARS-CoV-2 Omicron strain, and it is present in the specific sequence required for viroporin activity (61). The sequences that affect viroporin activity are N15 and V25 (16, 44, 57, 62). F20/F23/F26 and C40/C43 are conserved sequences among coronaviruses (20, 44, 54, 55), and F56/Y57/Y59 are sequences associated with E amyloidization (20, 59, 60). K63 is an amino acid involved in the RK/X/RK dibasic motif and has been suggested to function as an ER export signal sequence (20, 56). N66 is a putative glycosylation sequence (44, 55). D72/L73/L74/V75 is a motif sequence that binds to the PDZ domain protein present in host cells and is called the PBM (15, 42, 44). First, we confirmed the expression of each E mutant using fluorescence immunostaining and found that there were no significant changes in subcellular localization (Fig. 5G). Then, we expressed each E mutant and observed its effect on the secretion rate of VLPs (Fig. 5H through J). Compared to the wild-type E protein-expressing cells, the secretion rate of VLPs from cells expressing the F56A/Y57A/Y59A, N66A, and D72A/L73A/L74A/V75A mutations was dramatically reduced. These results suggest that the ability of the E-protein to perform its essential functions in VLP release is dependent on amyloidization, *N*-glycosylation, and the ability to bind host PDZ domain proteins.

### Inhibition of lysosomal acidification in SARS-CoV-2-infected cells

**Fig 6:**
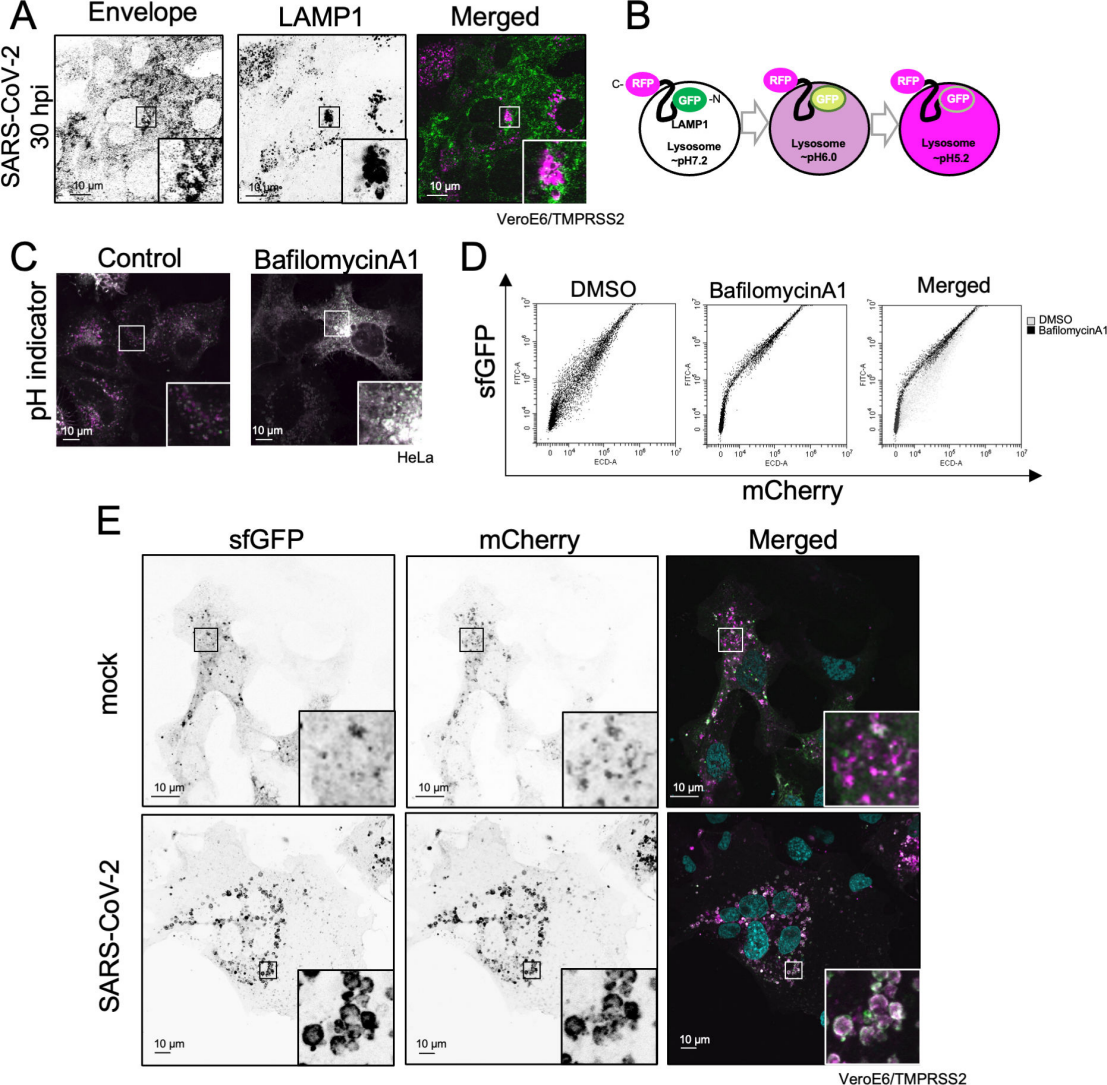
Inhibition of lysosomal acidification in SARS-CoV-2-infected cells. (**A**) Lysosomal localization of the E protein in SARS-CoV-2-infected cells. VeroE6/TMPRSS2 cells were infected with SARS-CoV-2 at MOI = 1.0. Thirty hours after infection, the cells were fixed and stained with anti-E (left panel, green) and anti-LAMP1 (middle panel, magenta) proteins, respectively. A merged image is shown in the right panel. Bar = 10 µm. (**B**) Schematic illustration of the pH indicator. (**C**) Comparative determination of lysosomal acidity using the pH indicator. HeLa cells expressing the pH indicator were treated with (right panel) or without (left panel) 50 nM bafilomycin A1 for 18 h. (**D**) Flow cytometry analysis of cells expressing the pH indicator. The HEK293T cells that expressed the pH indicator were treated with (middle panel) or without (left panel) 50 nM bafilomycin A1 for 18 h. Twenty-four hours post transfection, the cells were loaded into the flow cytometer. The *Y*-axis corresponds to sfGFP fluorescence measurements, and the *X*-axis corresponds to mCherry fluorescence measurements. Merged results are shown in the right panel (gray dots: control cells; black dots: BafA1-treated cells). (**E**) Lysosomal acidity of SARS-CoV-2-infected cells. VeroE6/TMPRSS2 cells expressing the pH indicator were infected with mock (upper panels) or SARS-CoV-2 (bottom panels) at MOI = 1.0. Twenty-four hours after infection, sfGFP (left panels) and mCherry (middle panels) signals were detected using a fluorescence microscope. Merged images are shown in the right panels. Magnified images are shown at the right bottom. Bar = 10 µm.

SARS-CoV-2 is known to be secreted through lysosomes, and β-CoV infection is known to raise the pH of lysosomes (19). To investigate the role of lysosomes in SARS-CoV-2 infection, we confirmed the co-localization of the E protein and LAMP1, a lysosome marker protein, during SARS-CoV-2 infection (Fig. 6A). Next, we measured lysosomal pH using a pH indicator to determine the effect of E protein localization on their acidity. A pH indicator was constructed by fusing sfGFP to the N-terminus of LAMP1 and mCherry to the C-terminus, as previously described (63). When a lysosome is functioning normally, sfGFP fluorescence is quenched due to the acidic environment and only mCherry signals are detected. However, both sfGFP and mCherry signals are detected when lysosomes are not properly acidified (Fig. 6B). Upon transfection into HeLa cells and observation under a fluorescence microscope, this pH indicator allowed the visualization of acidified lysosomes, which had only mCherry puncta (Fig. 6C, left). However, the addition of BafA1, which inhibits lysosomal acidification, resulted in the loss of mCherry puncta. This confirmed the utility of this pH indicator as a marker of lysosomal acidification (Fig. 6C, right). Furthermore, transfection of cells with the pH indicator and subsequent flow cytometry (FCM) analysis confirmed that sfGFP fluorescence decreased after DMSO treatment and increased after BafA1 treatment (Fig. 6D). After VeroE6/TMPRSS2 cells were infected with SARS-CoV-2, the expression of this pH indicator showed that the lysosomes underwent hypertrophy and deacidification (Fig. 6E). This indicates that SARS-CoV-2 infection causes lysosomal deacidification.

### The functional domain of the E protein is required for the inhibition of lysosome acidification

**Fig 7:**
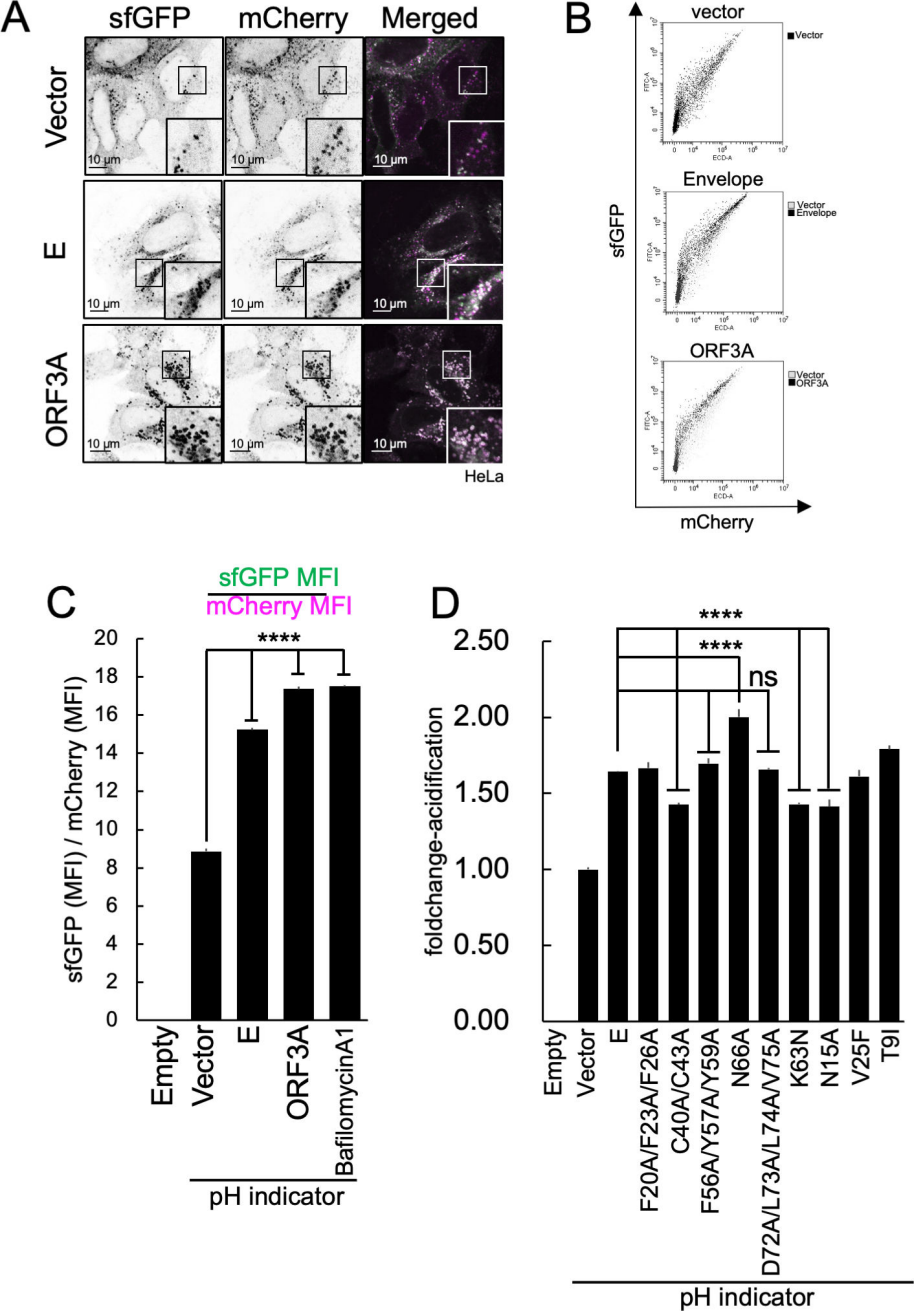
Functional domain of E protein required for the inhibition of lysosomal acidification. (**A**) Effect of E protein or ORF3A protein expression on lysosomal acidification. HeLa cells were co-transfected with an empty vector (top row), E- (middle row) or ORF3A-expressing vector (bottom row), and pH indicator-expressing vector. Twenty-four hours after transfection, the cells were observed and sfGFP (left column) and mCherry (middle column) signals were obtained using a fluorescence microscope. (**B**) Flow cytometry analysis of lysosomal acidification in cells expressing the E or ORF3A proteins. The HEK293T cells were co-transfected with an empty vector (top panel), E- (middle panel) or ORF3A-expressing vectors (bottom panel), and pH indicator-expressing vector. Twenty-four hours after transfection, the cells were harvested and the sfGFP (*Y*-axis) and mCherry (*X*-axis) signals were measured using a flow cytometer. (**C**) Quantification of the results from flow cytometry analysis in (**B**). The disappearance of the sfGFP signal was calculated by dividing the MFI value of the sfGFP MFI by that of mCherry. The results are shown as fold change values. *N* = 3. ****P < 0.0001; ns, not significant (Tukey’s *t-*test). (**D**) Effect of the expression of E mutants on lysosomal acidification. The HEK293T cells were co-transfected with an empty vector, wild-type E protein, or the indicated E mutant protein-expressing vectors. Twenty-four hours after transfection, the cells were harvested and analyzed using flow cytometry. The disappearance of the sfGFP signal was calculated using the same method described in (**C**). *N* = 3. ****P < 0.0001; ns, not significant (Tukey’s *t-*test).

We used a pH indicator to study the factors that contribute to lysosomal deacidification in SARS-CoV-2-infected cells. We co-expressed the ORF3A protein, which has been previously demonstrated to induce lysosomal deacidification (19), with this pH indicator and then analyzed the cells using fluorescence microscopy (Fig. 7A). The expression of the ORF3A protein alone resulted in lysosomal deacidification (Fig. 7A, bottom). The expression of ORF3A alone also resulted in lysosomal hypertrophy in cells. These results are in accordance with previous reports (19, 64). The SARS-CoV-2 E protein can form a pentamer and act as a viroporin (61). The E protein in SARS-CoV-1 also exhibits viroporin activity (28, 30), and the disruption of this activity affects its virulence (16). Therefore, the previously described pH indicator was used to examine how the viroporin activity of the E protein affected lysosomal pH. As shown in Fig. 7A, the expression of the E protein also resulted in an increase in lysosomal pH. The lysosomal acidification was then quantified using FCM analyses of cells that expressed the pH indicator and either the E or ORF3A protein (Fig. 7B). Lysosomal acidification was quantified using the mean fluorescence intensity (MFI) of sfGFP and mCherry obtained by FCM analyses of the cells. The sfGFP MFI of E- or ORF3A-expressing cells was significantly higher than that of the cells that expressed the empty vector (Fig. 7C). This indicates that the lysosomes were deacidified by the expression of the E or ORF3A proteins. In addition, we examined the effects of the previously described E mutant proteins on lysosomal acidification. We found that the C40/C43A, K63N, and N15A mutations inhibited E-induced lysosomal deacidification. These results suggest that these residues are critical for the ability of the E protein to deacidify lysosomes. However, the N66A mutation also facilitated lysosomal acidification. This result suggests that glycosylation site of E is controlled by its proper membrane topology and function (65). However, the F56A/Y57A/Y59A, N66A, and D72A/L73A/L74A/V75A mutant E proteins, which were able to inhibit VLP production, were unable to inhibit lysosomal acidification (Fig. 7D). This suggests that the E protein has two distinct functions: a lysosomal deacidification function required for the efficient extracellular release of virus particles and a function required for virus particle formation.

## DISCUSSION

In this study, we investigated the specific role of the E protein in virus particle formation and lysosomal deacidification. The relationship between these two roles of the E protein in SARS-CoV-2-infected cells has not yet been clarified. Because lysosomal deacidification enhances VLP release, it is unclear whether the E protein contributes to virus particle release through its role as a structural or non-structural viroporin protein. In this study, we found that these functions can be separated because they are determined by distinct motifs and that both functions driven by different functional domains collaboratively contribute to virus particle release.

Virus-like particles are vesicles composed of viral proteins that assemble when viral structural proteins are expressed (24). Electron microscopy of VLPs in cells and analyses using density gradient ultracentrifugation showed that the morphology of the VLPs is similar to that of the actual virions. Biosafety level 3 (BSL3) facilities are required for SARS-CoV-2 experiments, which has limited progress in understanding the molecular mechanisms of virus propagation. Our VLP production system can be used in BSL2 facilities and has the potential to be a useful tool for studying SARS-CoV-2 particle formation. In the past, research has mainly relied on western blotting to quantify the VLPs produced (18, 23, 24), but this method is not sensitive enough for high-throughput analysis. Our study utilized HiBiT-tags to label VLPs, which allows for more sensitive quantification than western blotting. In addition, HiBiT-based experiments can be conducted on a small scale. We believe that this high-sensitivity, high-throughput experimental system will be helpful in the effort to eradicate COVID-19.

The ESCRT pathway is generally known as the pathway that enveloped viruses such as retroviruses use for budding (46, 47). In our study, we discovered that inhibiting the ESCRT pathway did not prevent the release of VLPs (Fig. 3D through F) and the propagation of SARS-CoV-2 (Fig. 3G), indicating that SARS-CoV-2 may not rely on the ESCRT pathway for budding but instead may utilize the E and M proteins to manipulate membrane curvature. Further research is needed to confirm the ESCRT-independent mechanism of viral particle formation in SARS-CoV-2.

The E and M proteins are essential for the formation of virus particles, as shown by the significant inhibition of VLP formation in the absence of either protein (Fig. 2). The secretion of VLPs was also greatly affected by the addition of tags to the C-terminus of the E protein (Fig. 5C through E), suggesting that the structures of C-terminus and the factors that bind to them play crucial roles in E protein function. Our analysis of C-terminal mutants of the E protein revealed that substituting A for D72/L73/L74/V75, a PBM required for interaction with host PDZ domain proteins, significantly decreased VLP secretion (Fig. 5H through J). This indicates that the E-PDZ protein interaction is critical for the activity of the E protein in virion formation.

Previously, the C-terminal 55-SFYVYSRVK-63 sequence was identified as the oligomeric unit of the E protein (20, 59, 60). This motif, known as the amyloidization motif, can allow the formation of peptide aggregates *in vitro*. In this study, we generated a F56A/Y57A/Y59A mutant and found that this sequence is necessary for VLP formation but not for lysosomal deacidification (Fig. 5H through J and Fig. 7D). Although the induction of amyloidization in SARS-CoV-2-infected cells remains unknown, these properties of the E protein could aid its incorporation into virions and support virus particle formation.

The glycosylation of residue N66 has been previously reported (65). Intriguingly, the mutation of this residue resulted in higher molecular weight forms, similar to dimers and trimers, of the E protein (55), implying that the glycosylation of N66 could inhibit E protein oligomerization and support its specific role. Our results indicate that this mutation affects VLP formation (Fig. 5H through J) and suggests that proper E protein oligomerization is necessary for SARS-CoV-2 particle formation.

Previously, the SARS-CoV E protein was thought to be important for viral virulence but not essential for viral replication (17). Some studies have found that SARS-CoV-2 viruses lacking the E-ORF3A protein can still cause secondary infections even in the absence of E-ORF3A in their genome after multiple passages (66). Although these viruses have reduced infectious titers and pathogenicity, E-ORF3A may not be necessary for the formation of virus particles. However, our study revealed that the E protein is required for the efficient formation of virus particles (Fig. 2), indicating that the E protein plays a role in promoting virus particle formation.

It is also known that the E protein functions as a viroporin and allows the transport of cations such as Ca^2+^ and Na^+^ (67, 68). Our study, along with that of Xia et al., found that the ion channel activity of the E protein increased lysosomal pH (61). In addition to the E protein, the ORF3A protein exhibits viroporin activity (19, 69), suggesting that multiple viral factors may have redundant functions in ion channel activity. Although inhibiting the viroporin activity of the E protein has been shown to improve the pathology of SARS and reduce cytokine expression (16, 67), this may also affect viral replication in infected cells. The ability of the E viroporin to inhibit lysosomal acidification may prevent the degradation of virus particles and facilitate their efficient release.

The lysosomal deacidification caused by BafA1 increased the release of VLPs (Fig. 4A). It is possible that this deacidification helps protect SARS-CoV-2 particles from degradation in lysosomes as they are transported and released outside of the cell. In addition, our results indicate that the loss of the PBM function of the E protein did not affect the inhibition of lysosomal acidification (Fig. 7D). Previous studies have shown that SARS-CoV-1 and SARS-CoV-2 with PBM-deficient E proteins had significantly reduced infectious titers (15, 42), suggesting that this outcome might not be correlated with lysosomal function in host cells.

In this study, we discovered that E and ORF3A proteins share similar functions. Both are transmembrane proteins that can act as viroporins and participate in lysosomal deacidification, ultimately aiding in the release of progeny virions. However, in contrast to ORF3A, the coronavirus E protein is known to be incorporated into the virion during the assembly process (70). Consistent with this, our study demonstrated the incorporation of a small portion of the E protein into VLPs (Fig. 5F). These findings strongly suggest that the E protein may play a specific role in viral particle formation, distinct from that of ORF3A. Previous studies conducted on other betacoronaviruses have shown that deletion of the E protein gene leads to the production of viral particles with abnormal morphology. This suggests that the E protein is involved in the formation of membrane curvature during budding. In the case of SARS-CoV-2, it is plausible that the E protein may have a similar function, playing a role in membrane curvature formation during viral budding. Similar to the E protein, ORF3A possesses a PBM at its C-terminus. However, we have not examined the significance of ORF3A-PBM in lysosomal deacidification. Investigation of this aspect may offer insights into further functional differences between E and ORF3A proteins.

Our study revealed that residue N15 is necessary for E protein-mediated lysosomal deacidification (Fig. 7D). The N15 residue has previously been found to be crucial for viroporin formation due to its impact on the ion channel activity of the E protein (16). In this study, we found that the mutation in the V25 residue did not have a significant impact on the function of the E protein in lysosomal deacidification despite its known importance in ion channel activities. Previous studies assessing ion channel activities primarily focused on conductance, calcium, or sodium ions but not protons (57). Therefore, the role of the V25 residue may be specific to its interactions with specific substrates. Furthermore, it has been shown that mutations in C40/C43 caused lysosomal deacidification (Fig. 7D). This mutation may inhibit viroporin function by preventing the self-assembly of E proteins. A previous report showed that the coronavirus E protein does not form disulfide bonds (71). Other studies have shown that the C40/C43 residue of the E protein is involved in palmitoylation and self-assembly (55). Palmitoylation-mediated membrane associations and oligomer formation could be crucial for viroporin activity. This study also identified another essential residue, K63, for lysosome deacidification (Fig. 7D). The K63 residue is part of the RK/X/RK dibasic motif and is thought to play a role in ER export (20). Although there was no noticeable difference in the subcellular localization of the E protein mutants in our imaging analysis (Fig. 5G), the K63 mutation may affect the transport of the E protein from the ER to the lysosomes, resulting in impaired lysosomal function. Notably, the N15A, C40A/C43A, and K63N mutations, which affect the role of the E protein in lysosome deacidification, did not affect VLP release (Fig. 5J). These findings contradict the results presented in Fig. 4, which shows that VLP release was enhanced by lysosomal deacidification. This could be due to the fact that these mutations did not entirely impede lysosomal deacidification, as shown in Fig. 7D.

This study showed that interactions between the E protein and proteins with PDZ domains are crucial for the formation of VLPs (Fig. 5H through J). This indicates that VLP production requires interaction between the E protein and proteins with PDZ domains. The PDZ domain is known to be involved in various biological processes, including protein transport, cell adhesion, ion channel formation, and signal transduction (40). Previously, syntenin1 and PALS1 were identified as PDZ domain partners of the SARS-CoV-1 E protein. However, recent proteomic analyses suggest that various other PDZ domain proteins may also interact with the SARS-CoV-2 E protein (41). This suggests that cell type-specific partners may exist or that multiple factors may have redundant functions in E-protein-mediated particle formation. Currently, there are no drugs that specifically target the formation of SARS-CoV-2 virus particles. Our findings suggest that it may be possible to treat SARS-CoV-2 more effectively by using drugs that inhibit virion formation in combination with the currently available E-PDZ-inhibiting drugs. This approach may help increase the effectiveness of treatments for SARS-CoV-2 infections and potentially reduce disease severity.

## MATERIALS AND METHODS

### Cells, viruses, and reagents

The HEK293T, HeLa, and Vero E6/TMPRSS2 cells were cultured in Dulbecco’s modified Eagle’s medium supplemented with 10% fetal bovine serum (FBS), 1 mg/mL G418, 100 U/mL penicillin, and 100 µg/mL streptomycin at 5% CO_2_ and 37°C.

SARS-CoV-2 (OMC-510 isolate (72)) was propagated in Vero E6/TMPRSS2 cells. SARS-CoV-2 titrations were performed using a plaque-forming unit assay. Briefly, SARS-CoV-2 was sequentially diluted with the growth medium, and then, the dilution was added to confluent Vero E6/TMPRSS2 cells. After infection, the Vero E6/TMPRSS2 cells were cultured in modified Eagle’s medium supplemented with 2% FBS, 100 U/mL penicillin, 100 µg/mL streptomycin, and 1.0% (wt/vol) methylcellulose at 5% CO_2_ and 37°C. Three days post infection, the Vero E6/TMPRSS2 cells were fixed with formaldehyde and stained with crystal violet to visualize the plaques.

For the SARS-CoV-2 infection experiment, the virus was inoculated at a multiplicity of infection (MOI) = 1.0. Plasmids were transfected into HEK293T and HeLa cells using polyethylenimine (PEI)-Max (Polysciences, Warrington, PA, USA) or Lipofectamine 3000 (Thermo Fisher Scientific, Waltham, MA, USA), respectively, according to the manufacturer’s protocol. BafilomycinA1 (AdipoGen) was dissolved in DMSO. TritonX-100 (Nacalai Tesque, Kyoto, Japan) was dissolved in water at a concentration of 20% (wt/vol). The plasmids and antibodies used are listed in Tables 1 and 2.

**TABLE 1:** Antibody list

Name	Host	Clone #	Dilution factor	Conjugation	Experiment	Source
FLAG	Mouse	M2	1:1,000	None	WB, ICC	
Myc	Mouse	9E10	1:1,000	None	WB	Sigma
α-Tubulin	Mouse	DM1A	1:1,000	None	WB	Sigma
SARS-CoV-2 (COVID-19) spike S1	Rabbit	HL6	1:1,000	None	WB	GeneTex
SARS-CoV-2 (COVID-19) nucleocapsid	Rabbit		1:1,000	None	WB	GeneTex
Sars membrane	Rabbit		1:1,000	None	WB	Novus
SARS-CoV-2 (COVID-19) envelope	Rabbit	HL1443	1:500	None	ICC	GeneTex
SARS-CoV-2 ORF3a	Mouse	1035921	1:1,000	None	WB	R & D
LAMP1	Mouse	H4A3	1:1,000	None	ICC	Santa Cruz Biotechnology
Anti-VPS4A	Rabbit		1:1,000	None	WB	Original

**TABLE 2:** Plasmid list

Plasmid name	Backbone	Epitope tag	Selection
pCAG.MCS2	pCAG.MCS2		Amp
pCAG.Myc	pCAG.Myc		Amp
pCAG.MCS2-Myc	pCAG.MCS2-Myc		Amp
pcDNA3.1 MycHis(−)A	pcDNA3.1 MycHis(−)A	Myc	Amp
pEGFP-C1	pEGFP-C1	EGFP	Kan
pCAG.MCS2-FLAG-HiBiT	pCAG.MCS2-FLAG-HiBiT	FLAG, HiBiT	Amp
pCAG.HiBiT-FLAG	pCAG.HiBiT-FLAG-MCS2	FLAG, HiBiT	Amp
pCAG.SARS-CoV-2-S-FLAG-HiBiT	pCAG.MCS2-FLAG-HiBiT	FLAG, HiBiT	Amp
pCAG.SARS-CoV-2-M	pCAG.MCS2		Amp
pCAG.SARS-CoV-2-N	pCAG.MCS2		Amp
pCAG.SARS-CoV-2-E	pCAG.MCS2		Amp
pCAG.SARS-CoV-2-ORF3A	pCAG.MCS2		Amp
pCAG.SARS-CoV-2-E	pCAG.MCS2		Amp
pCAG.SARS-CoV-2-E-F20/F23/F26A	pCAG.MCS2		Amp
pCAG.SARS-CoV-2-E-C40/C43A	pCAG.MCS2		Amp
pCAG.SARS-CoV-2-E-F56/Y57/Y59A	pCAG.MCS2		Amp
pCAG.SARS-CoV-2-E-N66A	pCAG.MCS2		Amp
pCAG.SARS-CoV-2-E-D72/L73/L74/V75A	pCAG.MCS2		Amp
pCAG.SARS-CoV-2-E-K63N	pCAG.MCS2		Amp
pCAG.SARS-CoV-2-E-N15A	pCAG.MCS2		Amp
pCAG.SARS-CoV-2-E-V25F	pCAG.MCS2		Amp
pCAG.SARS-CoV-2-E-T9I	pCAG.MCS2		Amp
pCAG.SARS-CoV-2-Myc-E	pCAG.Myc	Myc	Amp
pCAG.SARS-CoV-2-E-Myc	pCAG.MCS2-Myc	Myc	Amp
pcDNA3.1-sfGFP-LAMP1-mCherry	pcDNA3.1 MycHis(−)A	sfGFP,mCherry	Amp
pEGFP-VPS4A K173Q	pEGFP-C1	EGFP	Kan
pQH-Gag	pQC-xIN	FLAG,HiBiT	Amp
hACE2	Addgene#1786		Amp

### Plasmid construction

To express the SARS-CoV-2 structural protein in mammalian cells, M, N, E, and ORF3A protein expression vectors were constructed by inserting their respective sequences into the KpnI/XhoI sites of pCAG.MCS2 (73) vectors using ligation. The S-FLAG-HiBiT was constructed by inserting its sequence into the KpnI/XhoI site of pCAG.MCS2-FLAG-HiBiT using ligation. The plasmids encoding E protein mutants were generated using the inverse PCR method with corresponding primer pairs. The pH indicator pcDNA3.1-sfGFP-LAMP1-mCherry was designed and constructed as previously described (63). Briefly, DNA fragments of the signal sequences of bovine prolactin and sfGFP were connected to the N-terminus of hLAMP1, and mCherry was then connected to the C-terminus of hLAMP1. These DNA fragments were amplified using PCR and the corresponding primer pairs. The resulting DNA fragments were then inserted into the NheI/XhoI site of pcDNA3.1 MycHis(−)A using ligation. All constructs were amplified by DH5α cells, and the DNA sequences were confirmed.

### SARS-CoV-2 VLP preparation, detection of HiBiT-dependent NanoLuc luciferase activity (HiBiT activity), and detergent assays

SARS-CoV-2 VLPs were produced using a previously described method (24). Briefly, HEK293T cells were transfected with structural protein expression vectors using PEI at a molar ratio of 8:6:3:8 for S:M:N:E or 8:6:3:8:6 for S:M:N:E:ORF3A. Twelve hours after transfection, the culture supernatant was changed. To observe the effect of bafilomycin A1, it was added to a final concentration of 50 nM at the time of medium exchange. Twenty-four hours after medium exchange, HEK293T cell culture supernatants were harvested and centrifuged (500 × *g* for 5 min; 1,200 × *g* for 5 min; 10,000 × *g* for 5  min) to remove cell debris. After centrifugation at 10,000 × *g*, the supernatant was ultracentrifuged (100,000 × *g* for 70 min) to precipitate SARS-CoV-2 VLPs. The VLP precipitates and cells were suspended in phosphate-buffered saline (PBS). The HiBiT tag in the VLPs or transfected cells was detected using a Nano-Glo HiBiT Lytic Detection System (Promega) and a Varioskan LUX Multimode microplate reader (Thermo Fisher Scientific) according to the manufacturer’s protocol. Equal volumes of Nano-Glo HiBiT Lytic Buffer containing a 1.0% LgBiT protein solution and 2.0% Nano-Glo HiBiT Lytic Substrate were added to the supernatant, PBS-suspended pellet, or cells. In a 384-well white-bottom assay plate (Greiner Bio-one, Kremsmünster, Austria), 10 µL of sample solution was mixed with 10 µL of reaction buffer. Luciferase signals were measured immediately after mixing, using a Varioskan LUX microplate reader (Thermo Fisher Scientific). For the detergent sensitivity assay, we used detergent sensitivity assay buffer (PBS containing a 1.0% LgBiT protein solution and 2.0% Nano-Glo HiBiT Lytic Substrate). The assay was conducted with or without the addition of 0.1% TritonX-100 (Nacalai, Kyoto, Japan).

### ESCRT pathway inhibition

To inhibit the ESCRT pathway, the VPS4A K173Q mutant (48) was co-expressed with the HiBiT-labeled VLP constructs. Inhibition of the ESCRT pathway was confirmed by examining HiBiT activity in the culture supernatants of cells expressing human codon-optimized HIV-1 Gag protein (74) fused with the HiBiT tag. To purify the Gag protein, we followed the methods outlined in a previous report (48). Briefly, the culture supernatants of HEK293T cells were harvested after 36 h of transfection and subjected to continuous centrifugation (500 × *g* for 5  min; 1,200 × *g* for 5  min; 20,000 × *g* for 90  min) to remove cell debris. After centrifugation, the pellet from the 20,000 × *g* centrifugation step was collected and resuspended in PBS. The HiBiT activity was measured using the method described above. For the ESCRT inhibition assay, VPS4A K173Q-expressing plasmids or empty plasmids were co-transfected with VLP-expressing vectors at a molar ratio of 1:1, and the HiBiT activities of the culture supernatant were measured 36 h post transfection.

### Western blotting

The cells were directly lysed in sodium dodecyl sulfate polyacrylamide gel electrophoresis (SDS-PAGE) sample buffer and boiled for 5 min at 95°C. The SDS-PAGE was performed using a 10% WIDE RANGE polyacrylamide gel (Nacalai Tesque, Kyoto, Japan). The proteins were electrically transferred to a polyvinylidene fluoride membrane (Immobilon-P; MilliporeSigma, Burlington, MA, USA), and the membranes were blocked using blocking buffer {3% skim milk, TBS-T [25 mM Tris pH 7.5], 137 mM NaCl, 0.27 mM KCl, 0.05% Tween20} for 30 min at 25°C. The membranes were incubated overnight at 4°C with diluted primary antibodies (see Table 1). After rinsing three times with TBS-T, the membrane was incubated with diluted secondary antibodies for 60 min at 25°C. After washing out the unbound secondary antibodies, the immunoreactive signals were detected using EzWestLumi plus (ATTO Technology, Amherst, NY, USA) and a chemiluminescence detector (iBRIGHT CL1000; Thermo Fisher Scientific).

### Immunofluorescence and fluorescence microscopy

For the analysis of cells expressing viral proteins, HeLa cells transfected with plasmid vectors were cultured on glass coverslips for 24 h and fixed with 4% paraformaldehyde in PBS (Nacalai Tesque) for 15 min. Then, the cells were permeabilized with PBS containing 0.1% Triton X-100 (vol/vol) for 10 min. For the analysis of SARS-CoV-2-infected cells, VeroE6/TMPRSS2 cells were infected with SARS-CoV-2 at MOI = 1.0, cultured for 30 h, and then fixed with 100% methanol for 6 min. After fixation and permeabilization, the cells were treated with blocking buffer [PBS containing 0.1% Triton X-100 (vol/vol) and 10% fetal bovine serum] for 30 min and incubated with diluted primary antibodies (see Table 1) for 60 min. After washing to remove the unbound primary antibodies, the cells were incubated with various Alexa fluor-conjugated secondary antibodies for 60 min. The cells were then mounted on coverslips using Fluoromount-G (SouthernBiotech). Fluorescent images were captured using an Olympus FV3000 laser-scanning confocal microscope (Olympus).

### Sucrose density gradient analysis

The purified SRAS-CoV-2 VLPs described above were loaded onto a 20%–60% (wt/vol) linear sucrose density gradient in PBS. Sucrose density gradient ultracentrifugation was performed for 3 h at 220,000 × *g* and 4°C. The gradient was fractionated into 32 fractions from the top, and HiBiT activity in each fraction was detected as described above.

### Transmission electron microscopy analysis

To observe purified VLPs, SARS-CoV-2 VLPs were purified from 10 cm dishes of HEK293T cells that produced VLPs, and the VLPs were further purified using serial centrifugation and sucrose density gradient ultracentrifugation as described above. The fractions that contained the VLPs were collected and dialyzed in PBS, and then, the VLPs were concentrated using ultracentrifugation at 100,000 × *g* for 70 min. The pellet was resuspended in PBS. The samples were applied to a glow-discharged carbon-coated copper grid (Nisshin-EM, Tokyo, Japan) and stained using an EM-stainer (Nisshin-EM). The air-dried sample grids were observed using a JEM-2100 electron microscope (JEOL, Tokyo, Japan) operating at 200 kV and a normal magnification of ×10,000, and images were obtained using an Orius SC200D CCD camera (JEOL).

The preparation of ultra-thin sections from VLP-producing cells for observation was performed as previously described (75). Briefly, the HEK293T cells expressing VLPs were pelleted and then fixed with a 2% glutaraldehyde solution in 0.15 M PBS (pH 7.2) at 4°C for 3 h. After five washes with the 0.15 M PBS, the cells were post-fixed with 1% osmium tetroxide in 0.15 M PBS for 2 h. The samples were dehydrated in ethanol and embedded in an epoxy resin (Nisshin EM, Tokyo, Japan). Ultrathin sections (70 nm) were prepared using a Reichert-Nissei ultramicrotome (ULTRACUT-N; Nissei Sangyo, Tokyo, Japan) and mounted on a nickel grid. The sections were double-stained with uranyl acetate and Reynolds’ Pb and observed under TEM and ET (HT7800 types, Hitachi, Tokyo, Japan). Micrographs were captured using a CCD camera (XR-81).

### Comparative determination of lysosomal acidity using a pH indicator

For the analysis of viral protein-expressing cells, HEK293T cells transfected with pcDNA3.1-sfGFP-LAMP1-mCherry (pH indicator) and empty, E protein-, or ORF3A protein-expressing vectors were cultured for 24 h. For the analysis of SARS-CoV-2-infected cells, VeroE6/TMPRSS2 cells transfected with pcDNA3.1-sfGFP-LAMP1-mCherry were infected with SARS-CoV-2 at MOI = 1.0 and then cultured for 24 h. The cells were harvested and analyzed for GFP and mCherry fluorescence using a flow cytometer (CytoFLEX S, Beckman Coulter). The MFI values for the GFP or mCherry signals were obtained from GFP- and mCherry-double-positive cell populations. Lysosomal acidity was determined by dividing the MFI of GFP by that of mCherry. For the imaging analysis, cells were fixed with 4% paraformaldehyde and observed by fluorescence microscopy (Olympus FV3000 laser scanning confocal microscope).

### Statistical analysis

All graphical values are represented as mean ± standard deviation. One-way analysis of variance with Tukey’s test was performed using GraphPad Prism software (version 7.0; GraphPad Software Inc., La Jolla, CA, USA) to compare each group with the control group.

## References

[1] V’kovski P , Kratzel A , Steiner S , Stalder H , Thiel V . 2021. Coronavirus biology and replication: implications for SARS-CoV-2. Nat Rev Microbiol 19:155–170. doi:10.1038/s41579-020-00468-6 33116300 PMC7592455

[2] Asselah T , Durantel D , Pasmant E , Lau G , Schinazi RF . 2021. COVID-19: discovery, diagnostics and drug development. J Hepatol 74:168–184. doi:10.1016/j.jhep.2020.09.031 33038433 PMC7543767

[3] Malik YA . 2020. Properties of coronavirus and SARS-CoV-2. Malays J Pathol 42:3–11. 32342926

[4] Zhou P , Yang X-L , Wang X-G , Hu B , Zhang L , Zhang W , Si H-R , Zhu Y , Li B , Huang C-L , Chen H-D , Chen J , Luo Y , Guo H , Jiang R-D , Liu M-Q , Chen Y , Shen X-R , Wang X , Zheng X-S , Zhao K , Chen Q-J , Deng F , Liu L-L , Yan B , Zhan F-X , Wang Y-Y , Xiao G-F , Shi Z-L . 2020. A pneumonia outbreak associated with a new coronavirus of probable bat origin. Nature 588:270–273. doi:10.1038/s41586-020-2951-z PMC7095418 32015507

[5] Ahmed SF , Quadeer AA , McKay MR . 2020. Preliminary identification of potential vaccine targets for the COVID-19 coronavirus (SARS-CoV-2) based on SARS-CoV immunological studies. Viruses 12:254. doi:10.3390/v12030254 32106567 PMC7150947

[6] Bracquemond D , Muriaux D . 2021. Betacoronavirus assembly: clues and perspectives for elucidating SARS-CoV-2 particle formation and egress. mBio 12:e0237121. doi:10.1128/mBio.02371-21 34579570 PMC8546641

[7] de Haan CAM , Rottier PJM . 2005. Molecular interactions in the assembly of coronaviruses. Adv Virus Res 64:165–230. doi:10.1016/S0065-3527(05)64006-7 16139595 PMC7112327

[8] Klein S , Cortese M , Winter SL , Wachsmuth-Melm M , Neufeldt CJ , Cerikan B , Stanifer ML , Boulant S , Bartenschlager R , Chlanda P . 2020. SARS-CoV-2 structure and replication characterized by in situ cryo-electron tomography. Nat Commun 11:5885. doi:10.1038/s41467-020-19619-7 33208793 PMC7676268

[9] Arya R , Kumari S , Pandey B , Mistry H , Bihani SC , Das A , Prashar V , Gupta GD , Panicker L , Kumar M . 2021. Structural insights into SARS-CoV-2 proteins. J Mol Biol 433:166725. doi:10.1016/j.jmb.2020.11.024 33245961 PMC7685130

[10] Masters PS . 2006. The molecular biology of coronaviruses. Adv Virus Res 66:193–292. doi:10.1016/S0065-3527(06)66005-3 16877062 PMC7112330

[11] Marcink TC , Kicmal T , Armbruster E , Zhang Z , Zipursky G , Golub KL , Idris M , Khao J , Drew-Bear J , McGill G , Gallagher T , Porotto M , des Georges A , Moscona A . 2022. Intermediates in SARS-CoV-2 spike-mediated cell entry. Sci Adv 8:eabo3153. doi:10.1126/sciadv.abo3153 35984891 PMC9390989

[12] Fehr AR , Perlman S . 2015 Coronaviruses: an overview of their replication and pathogenesis. Coronaviruses 1282:1. doi:10.1007/978-1-4939-2438-7 PMC4369385 25720466

[13] Lu S , Ye Q , Singh D , Cao Y , Diedrich JK , Yates JR 3rd , Villa E , Cleveland DW , Corbett KD . 2021. The SARS-CoV-2 nucleocapsid phosphoprotein forms mutually exclusive condensates with RNA and the membrane-associated M protein. Nat Commun 12:502. doi:10.1038/s41467-020-20768-y 33479198 PMC7820290

[14] Neuman BW , Kiss G , Kunding AH , Bhella D , Baksh MF , Connelly S , Droese B , Klaus JP , Makino S , Sawicki SG , Siddell SG , Stamou DG , Wilson IA , Kuhn P , Buchmeier MJ . 2011. A structural analysis of M protein in coronavirus assembly and morphology. J Struct Biol 174:11–22. doi:10.1016/j.jsb.2010.11.021 21130884 PMC4486061

[15] Honrubia JM , Gutierrez-Álvarez J , Sanz-Bravo A , González-Miranda E , Muñoz-Santos D , Castaño-Rodriguez C , Wang L , Villarejo-Torres M , Ripoll-Gómez J , Esteban A , Fernandez-Delgado R , Sánchez-Cordón PJ , Oliveros JC , Perlman S , McCray PB , Sola I , Enjuanes L . 2023. SARS-CoV-2-mediated lung edema and replication are diminished by cystic fibrosis transmembrane conductance regulator modulators. mBio 14:e0313622. doi:10.1128/mbio.03136-22 36625656 PMC9973274

[16] Nieto-Torres JL , DeDiego ML , Verdiá-Báguena C , Jimenez-Guardeño JM , Regla-Nava JA , Fernandez-Delgado R , Castaño-Rodriguez C , Alcaraz A , Torres J , Aguilella VM , Enjuanes L . 2014. Severe acute respiratory syndrome coronavirus envelope protein ion channel activity promotes virus fitness and pathogenesis. PLoS Pathog 10:e1004077. doi:10.1371/journal.ppat.1004077 24788150 PMC4006877

[17] DeDiego ML , Alvarez E , Almazán F , Rejas MT , Lamirande E , Roberts A , Shieh W-J , Zaki SR , Subbarao K , Enjuanes L . 2007. A severe acute respiratory syndrome coronavirus that lacks the E gene is attenuated in vitro and in vivo. J Virol 81:1701–1713. doi:10.1128/JVI.01467-06 17108030 PMC1797558

[18] Boson B , Legros V , Zhou B , Siret E , Mathieu C , Cosset F-L , Lavillette D , Denolly S . 2021. The SARS-CoV-2 envelope and membrane proteins modulate maturation and retention of the spike protein, allowing assembly of virus-like particles. J Biol Chem 296:100111. doi:10.1074/jbc.RA120.016175 33229438 PMC7833635

[19] Ghosh S , Dellibovi-Ragheb TA , Kerviel A , Pak E , Qiu Q , Fisher M , Takvorian PM , Bleck C , Hsu VW , Fehr AR , Perlman S , Achar SR , Straus MR , Whittaker GR , de Haan CAM , Kehrl J , Altan-Bonnet G , Altan-Bonnet N . 2020. β-coronaviruses use lysosomes for egress instead of the biosynthetic secretory pathway. Cell 183:1520–1535. doi:10.1016/j.cell.2020.10.039 33157038 PMC7590812

[20] Mukherjee S , Bhattacharyya D , Bhunia A . 2020. Host-membrane interacting interface of the SARS coronavirus envelope protein: Immense functional potential of C-terminal domain. Biophys Chem 266:106452. doi:10.1016/j.bpc.2020.106452 32818817 PMC7418743

[21] Park SH , Siddiqi H , Castro DV , De Angelis AA , Oom AL , Stoneham CA , Lewinski MK , Clark AE , Croker BA , Carlin AF , Guatelli J , Opella SJ . 2021. Interactions of SARS-CoV-2 envelope protein with amilorides correlate with antiviral activity. PLoS Pathog 17:e1009519. doi:10.1371/journal.ppat.1009519 34003853 PMC8184013

[22] Yuan Z , Hu B , Xiao H , Tan X , Li Y , Tang K , Zhang Y , Cai K , Ding B , Goff SP . 2022. The E3 ubiquitin ligase RNF5 facilitates SARS-CoV-2 membrane protein-mediated virion release. mBio 13. doi:10.1128/mbio.03168-21 PMC8805027 35100873

[23] Plescia CB , David EA , Patra D , Sengupta R , Amiar S , Su Y , Stahelin RV . 2021. SARS-CoV-2 viral budding and entry can be modeled using BSL-2 level virus-like particles. J Biol Chem 296:100103. doi:10.1074/jbc.RA120.016148 33214224 PMC7832013

[24] Xu R , Shi M , Li J , Song P , Li N . 2020. Corrigendum: construction of SARS-CoV-2 virus-like particles by mammalian expression system. Front Bioeng Biotechnol 8:1026. doi:10.3389/fbioe.2020.01026 32850726 PMC7409377

[25] Gourdelier M , Swain J , Arone C , Mouttou A , Bracquemond D , Merida P , Saffarian S , Lyonnais S , Favard C , Muriaux D . 2022. Optimized production and fluorescent labeling of SARS-CoV-2 virus-like particles. Sci Rep 12:14651. doi:10.1038/s41598-022-18681-z 36030323 PMC9419636

[26] Wang C , Zheng X , Gai W , Zhao Y , Wang H , Wang H , Feng N , Chi H , Qiu B , Li N , Wang T , Gao Y , Yang S , Xia X . 2017. MERS-CoV virus-like particles produced in insect cells induce specific humoural and cellular Imminity in rhesus macaques. Oncotarget 8:12686–12694. doi:10.18632/oncotarget.8475 27050368 PMC5355045

[27] Cao Y , Yang R , Wang W , Lee I , Zhang R , Zhang W , Sun J , Xu B , Meng X . 2020. Computational study of the ion and water Permeation and transport mechanisms of the SARS-CoV-2 pentameric E protein channel. Front Mol Biosci 7:565797. doi:10.3389/fmolb.2020.565797 33173781 PMC7538787

[28] Pervushin K , Tan E , Parthasarathy K , Lin X , Jiang FL , Yu D , Vararattanavech A , Soong TW , Liu DX , Torres J . 2009. Structure and inhibition of the SARS coronavirus envelope protein ion channel. PLoS Pathog 5:e1000511. doi:10.1371/journal.ppat.1000511 19593379 PMC2702000

[29] Cao Y , Yang R , Lee I , Zhang W , Sun J , Wang W , Meng X . 2021. Characterization of the SARS-CoV-2 E protein: sequence, structure, viroporin, and inhibitors. Protein Sci. 30:1114–1130. doi:10.1002/pro.4075 33813796 PMC8138525

[30] Torres J , Surya W , Li Y , Liu DX . 2015. Protein-protein interactions of viroporins in coronaviruses and paramyxoviruses: new targets for antivirals Viruses 7:2858–2883. doi:10.3390/v7062750 26053927 PMC4488717

[31] Breitinger U , Farag NS , Sticht H , Breitinger HG . 2022. Viroporins: structure, function, and their role in the life cycle of SARS-CoV-2. Int J Biochem Cell Biol 145:106185. doi:10.1016/j.biocel.2022.106185 35219876 PMC8868010

[32] Sarkar M , Etheimer P , Hannothiaux V , Saha S . 2022. SARS-CoV-2 viroporins: a multi-omics insight from nucleotides to amino acids. Appl Microbiol 2:572–593. doi:10.3390/applmicrobiol2030045

[33] Nardella C , Visconti L , Malagrinò F , Pagano L , Bufano M , Nalli M , Coluccia A , La Regina G , Silvestri R , Gianni S , Toto A . 2021. Targeting PDZ domains as potential treatment for viral infections, neurodegeneration and cancer. Biol Direct 16:15. doi:10.1186/s13062-021-00303-9 34641953 PMC8506081

[34] Caillet-Saguy C , Durbesson F , Rezelj VV , Gogl G , Tran QD , Twizere J-C , Vignuzzi M , Vincentelli R , Wolff N . 2021. Host PDZ-containing proteins targeted by SARS-CoV-2. FEBS J. 288:5148–5162. doi:10.1111/febs.15881 33864728 PMC8250131

[35] Javorsky A , Humbert PO , Kvansakul M . 2021. Structural basis of coronavirus E protein interactions with human PALS1 PDZ domain. Commun Biol 4:724. doi:10.1038/s42003-021-02250-7 34117354 PMC8196010

[36] Shepley-McTaggart A , Sagum CA , Oliva I , Rybakovsky E , DiGuilio K , Liang J , Bedford MT , Cassel J , Sudol M , Mullin JM , Harty RN . 2021. SARS-CoV-2 envelope (E) protein interacts with PDZ-domain-2 of host tight junction protein ZO1. PLoS One 16:e0251955. doi:10.1371/journal.pone.0251955 34106957 PMC8189464

[37] Schoeman D , Cloete R , Fielding BC . 2022. The flexible, extended coil of the PDZ-binding motif of the three deadly human coronavirus E proteins plays a role in pathogenicity. Viruses 14:1707. doi:10.3390/v14081707 36016329 PMC9416557

[38] Toto A , Ma S , Malagrinò F , Visconti L , Pagano L , Stromgaard K , Gianni S . 2020. Comparing the binding properties of peptides mimicking the envelope protein of SARS-CoV and SARS-CoV-2 to the PDZ domain of the tight junction-associated PALS1 protein. Protein Sci. 29:2038–2042. doi:10.1002/pro.3936 32822073 PMC7461438

[39] Chai J , Cai Y , Pang C , Wang L , McSweeney S , Shanklin J , Liu Q . 2021. Structural basis for SARS-CoV-2 envelope protein recognition of human cell junction protein PALS1. Nat Commun 12:3433. doi:10.1038/s41467-021-23533-x 34103506 PMC8187709

[40] Lee HJ , Zheng JJ . 2010. PDZ domains and their binding partners: structure, specificity, and modification. Cell Commun Signal 8:8. doi:10.1186/1478-811X-8-8 20509869 PMC2891790

[41] Pearson GJ , Broncel M , Snijders AP , Carlton JG . 2021. Exploitation of the secretory pathway by SARS-CoV-2 envelope. bioRxiv. doi:10.1101/2021.06.30.450614

[42] Jimenez-Guardeño JM , Nieto-Torres JL , DeDiego ML , Regla-Nava JA , Fernandez-Delgado R , Castaño-Rodriguez C , Enjuanes L , Basler CF . 2014. The PDZ-binding motif of severe acute respiratory syndrome coronavirus envelope protein is a determinant of viral pathogenesis. PLoS Pathog 10:e1004320. doi:10.1371/journal.ppat.1004320 25122212 PMC4133396

[43] Schwinn MK , Machleidt T , Zimmerman K , Eggers CT , Dixon AS , Hurst R , Hall MP , Encell LP , Binkowski BF , Wood KV . 2018. CRISPR-mediated tagging of endogenous proteins with a luminescent peptide. ACS Chem Biol 13:467–474. doi:10.1021/acschembio.7b00549 28892606

[44] Schoeman D , Fielding BC . 2019. Coronavirus envelope protein: current knowledge. Virol J 16:69. doi:10.1186/s12985-019-1182-0 31133031 PMC6537279

[45] Ortego J , Escors D , Laude H , Enjuanes L . 2002. Generation of a replication-competent, propagation-deficient virus vector based on the transmissible gastroenteritis coronavirus genome. J Virol 76:11518–11529. doi:10.1128/jvi.76.22.11518-11529.2002 12388713 PMC136772

[46] Morita E. , Sundquist WI . 2004. Retrovirus budding. Annu Rev Cell Dev Biol 20:395–425. doi:10.1146/annurev.cellbio.20.010403.102350 15473846

[47] Morita E , Sandrin V , McCullough J , Katsuyama A , Baci Hamilton I , Sundquist WI . 2011. ESCRT-III protein requirements for HIV-1 budding. Cell Host Microbe 9:235–242. doi:10.1016/j.chom.2011.02.004 21396898 PMC3070458

[48] Garrus JE , von Schwedler UK , Pornillos OW , Morham SG , Zavitz KH , Wang HE , Wettstein DA , Stray KM , Côté M , Rich RL , Myszka DG , Sundquist WI . 2001. Tsg101 and the vacuolar protein sorting pathway are essential for HIV-1 budding. Cell 107:55–65. doi:10.1016/s0092-8674(01)00506-2 11595185

[49] Zeng C , Evans JP , King T , Zheng Y-M , Oltz EM , Whelan SPJ , Saif LJ , Peeples ME , Liu S-L . 2022. SARS-CoV-2 spreads through cell-to-cell transmission. Proc Natl Acad Sci U S A 119:e2111400119. doi:10.1073/pnas.2111400119 34937699 PMC8740724

[50] Trivedi PC , Bartlett JJ , Pulinilkunnil T . 2020. Lysosomal biology and function: modern view of cellular debris bin. Cells 9:5. doi:10.3390/cells9051131 PMC7290337 32375321

[51] Wang R , Wang J , Hassan A , Lee CH , Xie XS , Li X . 2021. Molecular basis of V-ATPase inhibition by bafilomycin A1. Nat Commun 12:1–8. doi:10.1038/s41467-021-22111-5 33741963 PMC7979754

[52] Yue Y , Nabar NR , Shi C-S , Kamenyeva O , Xiao X , Hwang I-Y , Wang M , Kehrl JH . 2018. SARS-coronavirus open reading frame-3A drives multimodal necrotic cell death. Cell Death Dis 9:1–15. doi:10.1038/s41419-018-0917-y 30185776 PMC6125346

[53] Bianchi M , Benvenuto D , Giovanetti M , Angeletti S , Ciccozzi M , Pascarella S . 2020. SARS-CoV-2 envelope and membrane proteins: structural differences linked to virus characteristics Biomed Res Int 2020:4389089. doi:10.1155/2020/4389089 32596311 PMC7261327

[54] Lopez LA , Riffle AJ , Pike SL , Gardner D , Hogue BG . 2008. Importance of conserved cysteine residues in the coronavirus envelope protein. J Virol 82:3000–3010. doi:10.1128/JVI.01914-07 18184703 PMC2258990

[55] Kuzmin A , Orekhov P , Astashkin R , Gordeliy V , Gushchin I . 2022. Structure and dynamics of the SARS-CoV-2 envelope protein monomer. Proteins 90:1102–1114. doi:10.1002/prot.26317 35119706

[56] Lim KP , Liu DX . 2001. The missing link in Coronavirus assembly: retention of the avian coronavirus infectious bronchitis virus envelope protein in the pre-golgi compartments and physical interaction between the envelope and membrane proteins. J Biol Chem 276:17515–17523. doi:10.1074/jbc.M009731200 11278557 PMC7982318

[57] Torres J , Maheswari U , Parthasarathy K , Ng L , Liu DX , Gong X . 2007. Conductance and amantadine binding of a pore formed by a lysine-flanked transmembrane domain of SARS coronavirus envelope protein. Protein Sci 16:2065–2071. doi:10.1110/ps.062730007 17766393 PMC2206980

[58] Li S , Yuan L , Dai G , Chen RA , Liu DX , Fung TS . 2019. Regulation of the ER stress response by the ion channel activity of the infectious bronchitis coronavirus envelope protein modulates virion release, apoptosis, viral fitness, and pathogenesis. Front Microbiol 10:3022. doi:10.3389/fmicb.2019.03022 32038520 PMC6992538

[59] Ghosh A , Pithadia AS , Bhat J , Bera S , Midya A , Fierke CA , Ramamoorthy A , Bhunia A . 2015. Self-assembly of a nine-residue amyloid-forming peptide fragment of SARS corona virus E-protein: mechanism of self aggregation and amyloid-inhibition of hIAPP. Biochemistry 54:2249–2261. doi:10.1021/acs.biochem.5b00061 25785896 PMC4903029

[60] Ghosh A , Bhattacharyya D , Bhunia A . 2018. Structural insights of a self-assembling 9-residue peptide from the C-terminal tail of the SARS corona virus E-protein in DPC and SDS micelles: a combined high and low resolution spectroscopic study. Biochim Biophys Acta Biomembr 1860:335–346. doi:10.1016/j.bbamem.2017.10.015 29038024 PMC7094419

[61] Xia B , Wang Y , Pan X , Cheng X , Ji H , Zuo X , Jiang H , Li J , Gao Z . 2022. Why is the SARS-CoV-2 omicron variant milder Innovation (Camb) 3:100251. doi:10.1016/j.xinn.2022.100251 35497020 PMC9040510

[62] Verdiá-Báguena C , Nieto-Torres JL , Alcaraz A , DeDiego ML , Torres J , Aguilella VM , Enjuanes L . 2012. Coronavirus E protein forms ion channels with functionally and structurally-involved membrane lipids. Virology 432:485–494. doi:10.1016/j.virol.2012.07.005 22832120 PMC3438407

[63] Webb BA , Aloisio FM , Charafeddine RA , Cook J , Wittmann T , Barber DL . 2021. pHLARE: a new biosensor reveals decreased lysosome pH in cancer cells. Mol Biol Cell 32:131–142. doi:10.1091/mbc.E20-06-0383 33237838 PMC8120692

[64] Miao G , Zhao H , Li Y , Ji M , Chen Y , Shi Y , Bi Y , Wang P , Zhang H . 2021. ORF3A of the COVID-19 virus SARS-CoV-2 blocks HOPS complex-mediated assembly of the SNARE complex required for autolysosome formation. Dev Cell 56:427–442. doi:10.1016/j.devcel.2020.12.010 33422265 PMC7832235

[65] Yuan Q , Liao Y , Torres J , Tam JP , Liu DX . 2006. Biochemical evidence for the presence of mixed membrane topologies of the severe acute respiratory syndrome coronavirus envelope protein expressed in mammalian cells. FEBS Lett 580:3192–3200. doi:10.1016/j.febslet.2006.04.076 16684538 PMC7094218

[66] Zhang X , Liu Y , Liu J , Bailey AL , Plante KS , Plante JA , Zou J , Xia H , Bopp NE , Aguilar PV , Ren P , Menachery VD , Diamond MS , Weaver SC , Xie X , Shi P-Y . 2021. A trans-complementation system for SARS-CoV-2 recapitulates authentic viral replication without virulence. Cell 184:2229–2238. doi:10.1016/j.cell.2021.02.044 33691138 PMC7901297

[67] Xia B , Shen X , He Y , Pan X , Liu F-L , Wang Y , Yang F , Fang S , Wu Y , Duan Z , Zuo X , Xie Z , Jiang X , Xu L , Chi H , Li S , Meng Q , Zhou H , Zhou Y , Cheng X , Xin X , Jin L , Zhang H-L , Yu D-D , Li M-H , Feng X-L , Chen J , Jiang H , Xiao G , Zheng Y-T , Zhang L-K , Shen J , Li J , Gao Z . 2021. SARS-CoV-2 envelope protein causes acute respiratory distress syndrome (ARDS)-Like pathological damages and constitutes an antiviral target. Cell Res 31:847–860. doi:10.1038/s41422-021-00519-4 34112954 PMC8190750

[68] McClenaghan C , Hanson A , Lee SJ , Nichols CG . 2020. Coronavirus proteins as ion channels: current and potential research. Front Immunol 11:573339. doi:10.3389/fimmu.2020.573339 33154751 PMC7586316

[69] Castaño-Rodriguez C , Honrubia JM , Gutiérrez-Álvarez J , DeDiego ML , Nieto-Torres JL , Jimenez-Guardeño JM , Regla-Nava JA , Fernandez-Delgado R , Verdia-Báguena C , Queralt-Martín M , Kochan G , Perlman S , Aguilella VM , Sola I , Enjuanes L . 2018. Role of severe acute respiratory syndrome coronavirus viroporins E, 3a, and 8a in replication and pathogenesis. mBio 9:e02325-17. doi:10.1128/mBio.02325-17 29789363 PMC5964350

[70] Schoeman D , Fielding BC . 2019. Coronavirus envelope protein: current knowledge. Virol J 16:69. doi:10.1186/s12985-019-1182-0 31133031 PMC6537279

[71] Lee C , Yoo D . 2005. Cysteine residues of the porcine reproductive and respiratory syndrome virus small envelope protein are non-essential for virus infectivity. J Gen Virol 86:3091–3096. doi:10.1099/vir.0.81160-0 16227232

[72] Suzuki Y , Hishiki T , Emi A , Sakaguchi S , Itamura R , Yamamoto R , Matsuzawa T , Shimotohno K , Mizokami M , Nakano T , Yamamoto N . 2021. Strong alkaline electrolyzed water efficiently inactivates SARS-CoV-2, other viruses, and gram-negative bacteria. Biochem Biophys Res Commun 575:36–41. doi:10.1016/j.bbrc.2021.08.048 34455219 PMC8381626

[73] Morita E , Sandrin V , Chung H-Y , Morham SG , Gygi SP , Rodesch CK , Sundquist WI . 2007. Human ESCRT and ALIX proteins interact with proteins of the midbody and function in cytokinesis. EMBO J 26:4215–4227. doi:10.1038/sj.emboj.7601850 17853893 PMC2230844

[74] Kotsopoulou E , Kim VN , Kingsman AJ , Kingsman SM , Mitrophanous KA . 2000. A rev-independent human immunodeficiency virus type 1 (HIV-1)-based vector that exploits a codon-optimized HIV-1 gag-pol gene. J Virol 74:4839–4852. doi:10.1128/jvi.74.10.4839-4852.2000 10775623 PMC112007

[75] Wu H , Fujioka Y , Sakaguchi S , Suzuki Y , Nakano T . 2022. Three-dimensional reconstruction by electron tomography for the application to ultrastructural analysis of SARS-CoV-2 particles. Med Mol Morphol 55:60–67. doi:10.1007/s00795-021-00309-2 34825978 PMC8617558

